# The effects of twenty-four nutrients and phytonutrients on immune system function and inflammation: A narrative review

**Published:** 2021-05-27

**Authors:** Jillian Poles, Elisa Karhu, Megan McGill, H. Reginald McDaniel, John E. Lewis

**Affiliations:** ^1^Department of Exercise and Sport Science, University of North Carolina at Chapel Hill, Chapel Hill, NC, USA; ^2^Department of Medicine, University of Miami Miller School of Medicine, Miami, FL, USA; ^3^Department of Internal Medicine, Mount Sinai Medical Center Miami Beach, FL, USA; ^4^Wellness Quest, LLC, Grand Prairie, TX, USA; ^5^Department of Psychiatry and Behavioral Sciences, University of Miami Miller School of Medicine, Miami, FL, USA

**Keywords:** immune function, immunomodulation, inflammation, nutrients, phytonutrients

## Abstract

**Background and Aim:**

Recently, optimal immune function has become a primary focus of worldwide attention not only in the prevention of chronic disease but also as one strategy to reduce the severity of acute illness. Inflammation, a process largely controlled by the immune system, has long been studied and recognized for its role in chronic disease. Optimizing immune function or managing inflammation using individual nutrients and phytonutrients is not well understood by the average person. Thus, this narrative literature review summarizes many of the more recent findings about how certain nutrients and phytonutrients affect immune function and inflammation, and how they may best be utilized considering the growing worldwide interest in this topic.

**Methods:**

A comprehensive literature search of PubMed was performed to find clinical trials in humans that assessed the effect of nutrients and phytonutrients on immune function and inflammation, in individuals with acute and chronic health conditions, published in English between 2000 and 2020. Two independent reviewers evaluated the articles for their inclusion.

**Results:**

Eighty-seven articles were summarized in this narrative review. In total 24 nutrients and phytonutrients were included in the study, that is, acetyl-L-carnitine, Aloe vera polysaccharides, beta-glucans, bilberry, black seed oil, coenzyme Q10, curcumin (turmeric), frankincense, garlic, ginger, hydrolyzed rice bran, isoflavones, lipoic acid, mistletoe, N-acetyl cysteine, omega-3 fatty acids, resveratrol, selenium, shiitake mushroom and its derivatives, Vitamin B12, Vitamin C, Vitamin D3 (cholecalciferol), Vitamin E (d-alpha- and gamma-tocopherol), and zinc. Some of the noteworthy immune function and anti-inflammatory responses to these interventions included modulation of nuclear factor-Kappa B, tumor necrosis factor-a, interferon-g, interleukin-6, and CD4+ T cells, among others. These findings are not completely consistent or ubiquitous across all patient populations or health status.

**Conclusions:**

Based on this review, many nutrients and phytonutrients are capable of significantly modulating immune function and reducing inflammation, according to multiple biomarkers in clinical trials in different populations of adults with varying health statuses. Thus, dietary supplementation may serve as an adjunct to conventional pharmaceutical or medical therapies, but evaluation of risks and benefits for each person and health status is necessary. Additional larger studies are also needed to investigate the safety and efficacy of nutritional compounds in various health conditions, with emphases on potential drug-supplement interactions and clinical endpoints.

**Relevance for Patients:**

As demonstrated in the reviewed clinical trials, patients of various health challenges with a wide range of severity may benefit from select nutrients and phytonutrients to improve their immune function and reduce inflammation.

## 1. Introduction

With an underpinning in chronic inflammation, cardiovascular disease and cancer continue to be the leading causes of death worldwide [1]. The interest is rising in the average person to understand how to prevent these and other chronic diseases, not the least of which is by reducing inflammation. Furthermore, with the recent SARS-CoV-2 pandemic and intermittent concerns about infectious diseases, the immune system’s importance to health is becoming ever apparent and paramount to the average consumer. While acute inflammation is a necessary facet of basic immune function during processes of healing and combatting illness, chronic low-grade inflammation is associated with several autoimmune and metabolic conditions and can be detrimental to human health, shortening the lifespan, and impairing quality of life. Thus, how to improve the immune system’s functioning and capacity, along with reducing chronic inflammation, is now a leading question. Besides physical exercise, limiting or avoiding alcohol and tobacco, maintaining a healthy weight, and getting enough sleep, nutritional science is an evolving field in its application to immune system function, inflammation, and general health, in both preventative and restorative study models.

Dietary supplements offer one approach to adding key nutrients and phytonutrients to one’s daily food and drink consumption. Historically, nutrients, such as Vitamins C and D and zinc, have been studied for their effects on immune health, the incidence of colds, viral infections, and bacterial infections, and their anti-inflammatory activities. Phytonutrients, such as aloe polysaccharides, curcumin, genistein, lentinan, and rice bran arabinoxylan complex, have also been evaluated for their efficacy on immune functioning and immunomodulation. This review summarizes the recent findings for these nutrients and phytonutrients and assesses whether they offer the potential for helping a burgeoning audience of people who not only want to treat chronic disease and infection, but also to prevent such issues in the first place by maintaining a healthy and surveillant immune system. Given the recent rapid increase in the incidence of chronic diseases with downstream effects such as low-level chronic inflammation, this summary should be of interest to lay people (patients and their caregivers), clinicians, and researchers alike.

## 2. Methods

A comprehensive search for articles was performed using PubMed. Articles published in English between 2000 and 2020 with full text available were searched using the name of the nutrient or phytonutrient and the terms “immune function” OR “immune system” AND/OR “inflammation.” Inclusion criteria were: (a) Study published within the past 20 years, (b) a clinical trial conducted in humans, and (c) subjects had some sort of acute or chronic health condition. Two independent reviewers evaluated the articles for inclusion in the review. The search returned 1684 total articles, of which 87 were included in this review. The exclusion criteria for the other 1597 articles were: (a) Published before 2000, (b) written in a language other than English, (c) reporting on a study that was conducted in cells, tissues, or animals or an epidemiological or observational study in humans, (d) conducted in healthy individuals, or (e) another review paper. See Table 1 for a summary of the most significant effects of the nutrient or phytonutrient on immune function and inflammation according to the treatment amount, population under study, and sample size.

**Table 1:** Summary of primary effects of nutrients and phytonutrients on immune function and inflammation in clinical trials

Nutrient or phytonutrient	Study authors	Study population	Sample size (*n*)	Daily dose	Main immune results
**Antioxidant nutrients**					
Acetyl-L-carnitine	Milazzo *et al*., 2010 [6]	HIV+ with lipodystrophy	Treatment: 21 Placebo: 20	2 g/day of acetyl-L-carnitine for 48 weeks	Increased mitochondrial DNA in CD4+ T cells indicating a protective effect on mitochondrial function and immune function acetyl-L-carnitine
Coenzyme Q10 (ubiquinol)	Sanoobar *et al*., 2015 [10]	MS	Treatment: 24 Placebo: 24	500 mg/day of CoQ10 for 12 weeks	MMP-9, TNF-α, and IL-6 decreased significantly
	Cordero *et al*., 2014 [11]	Fibromyalgia	Treatment: 30 Placebo: 20	300 mg/day of CoQ10 divided into three dose for 40 days	NLRP3 and IL-1β gene expression were downregulated with a concurrent decrease in serum IL-1β and IL-18 levels
	Perez-Sanchez *et al*., 2017 [12]	Antiphospholipid Syndrome	Treatment: 36	200 mg/day reduced CoQ10 for 1 month	IL-8, VEGF, MIP-1α, IL-1β, and TNF-α levels in monocytes, oxidized LDL, and plasma VCAM-1 decreased
Lipoic acid	Jariwalla *et al*., 2008 [13]	HIV+ men and women, non-responsive to HARRT	Treatment: 15 Placebo: 18	300 mg α-lipoic acid 3 times daily for 6 months	Lymphocytes proliferated, blood glutathione level increased, but no changes in CD4+ and CD8+ T cell counts
	Sola *et al*., 2005 [14]	MetSyn	Treatment: 15 Placebo: 14	300 mg/day of α-lipoic acid for 4 weeks	IL-6 (15%) and PAI-1 (14%) decreased
	Yadav *et al*., 2005 [15]	MS	1200 mg: 7 1200 mg and Placebo: 9 2400 mg: 8 Placebo: 9	One of three different doses of lipoic acid: 600 mg twice/day, 1200 mg in the morning and placebo in the evening, or 1200 mg twice/day	MMP-9 and ICAM-1 activity reduced
N-acetyl cysteine	De Rosa *et al*., 2000 [16]	HIV	Treatment: 41 Placebo: 40	8000 mg/day of NAC	Whole blood glutathione and glutathione produced by T cells significantly increased, along with improved 2-3 years of survival
	Breitkreutz *et al*., 2000 [17]	HIV	Treatment: 21 Placebo: 16	600 mg/day of NAC for 180 days	CD4+ T cell count significantly increased at 60 days, and the decrease in viral load was attributed to ART, but was amplified by NAC
	Lai *et al*., 2012 [18]	Lupus	1.2 g: 9 2.4 g: 9 4.8 g: 9 Placebo: 9	One of three doses of NAC (1.2, 2.4, or 4.8 g/day) for 3 months	Peripheral blood lymphocytes, whole blood glutathione, mitochondrial mass, and spontaneous apoptosis rate in CD4-/CD8- double-negative T cells increased, mTOR activity significantly decreased, the SLEDAI significantly improved, 2.4 g/day was well-tolerated, while reversible nausea occurred in about one-third of the patients taking 4.8 g/day
	Tirouvanziam *et al*., 2006 [19]	Pediatric cystic fibrosis	Treatment: 18 Placebo: 9	Three doses of NAC: 0.6, 0.8, or 1.0 g 3 times/day for 4 weeks	Whole blood and neutrophil levels of glutathione significantly increased, and airway neutrophil count and sputum IL-8 level significantly reduced
	Purwanto *et al*., 2012 [20]	CKD patients receiving continuous ambulatory peritoneal dialysis	Treatment: 16 Placebo: 16	1.2 g/day of NAC for 8 weeks	Procalcitonin, IL-6, IL-1, and complement component 3 significantly decreased
	Kasperczyk *et al*., 2014 [21]	Lead-exposed workers	Treatment: 122 Placebo: 49	200, 400, or 800 mg/day of NAC for 12 weeks	Blood lead concentration significantly decreased in all groups, erythrocyte SOD activity significantly decreased in the 200 and 400 mg groups, erythrocyte glutathione peroxidase activity significantly decreased in the 200 and 800 mg groups, leukocyte MDA level significantly decreased in the 200 mg group, serum MDA level in the 400 mg group, and both serum and leukocyte MDA levels in the 800 mg group
	Csontos *et al*., 2011 [22]	Severe burn patients	Treatment: 14 Placebo: 14	150 mg/kg bolus of NAC followed by continuous administration of NAC for 6 days	Granulocyte CD11a and CD18 on days 4-6, granulocyte CD97 on days 2-6, and granulocyte CD49d on day 2 were lower, lymphocyte CD11a and CD49d on days 3-6 were significantly lower, and procalcitonin was significantly lower on days 2-4
Selenium	Hurwitz *et al*., 2007 [23]	HIV	Treatment: 141 Placebo: 121	200 µg/day of high selenium yeast for 9 months	An increase in serum selenium concentration was significantly negatively associated with the change in viral load, which was negatively associated with CD4+ T cell count, when controlling for the pre-treatment value
	Kamwesiga *et al*., 2015 [24]	HIV+, ART-naive	Treatment: 151 Placebo: 149	200 µg/day of selenium for 24 months	Significantly reduced rate of CD4+ T cell depletion, but was not significant enough to conclude a treatment effect on viral burden
	Faure *et al*., 2004 [25]	Type 2 diabetes	Treatment: 21 Placebo: 27	960 µg/day of selenium for 3 months	Plasma selenium and activity of selenium-dependent glutathione peroxidase were significantly increased and NF-κB activity was significantly decreased
	Hussain *et al*., 2019 [26]	Pulmonary tuberculosis	Treatment: 40 Placebo: 40	100 µg/day of selenium for 6-8 months	Lower leukocyte counts and IgG and IgM significantly increased, CD4+ and CD8+ T cells significantly increased, and MDA was significantly lower, while SOD, catalase, glutathione, and total antioxidants were higher
	Jahnova *et al*., 2002 [27]	Corticoid-dependent asthmatics	Treatment: 20	200 µg/day selenium for 6 months	PBMC expression of CD11a, CD11b, and CD62L significantly decreased with significant decreases in human umbilical vein endothelial cell expression of ICAM-1, VCAM-1, P-selectin, and E-selectin
Vitamin C (ascorbate)	Chien *et al*., 2004 [28]	Hyperlipidemic and uremic patients receiving hemodialysis regularly	Hyperlipidemic: 49 Uremic: 39 Control: 10	2.5 g Vitamin C in 150 mL of 5% albumin solution during their hemodialysis session for 6 months	Hydrogen peroxide levels reduced by 80-93%, the percentage of intracellular ROS production of polymorphonuclear neutrophils was significantly lower, and CRP significantly decreased
Vitamin E (d-alpha- and gamma-tocopherol)	Devaraj *et al*., 2000 [29]	Type 2 diabetes with and without macrovascular disease	With macrovascular disease: 25 Without: 25 Placebo: 25	1200 IU/day of alpha-tocopherol for 3 months	Superoxide anion, IL-1β, and TNF-α release (with greater inhibition of IL-1β than TNF-α), monocyte-endothelium adhesion in HUVECs, and sCAMs significantly decreased
	Devaraj *et al*., 2000 [30]	Type 2 diabetes with and without macrovascular disease	With macrovascular disease: 23 Without: 24 Placebo: 25	1200 IU/day of alpha-tocopherol for 3 months	CRP and monocyte IL-6 production significantly decreased
	Van Tits *et al*., 2000 [31]	Normotriglyceridemic and hypertriglyceridemic individuals	Hyperlipidemic: 12 Normolipidemic: 8	600 IU/day of alpha-tocopherol for 6 weeks	PMA-stimulated superoxide anion production and LPS-stimulated cytokine release were decreased in both normotriglyceridemic and hypertriglyceridemic individuals
	De Souza Junior *et al*., 2005 [32]	HIV+ on ART	Treatment: 14 Placebo: 15	800 mg/day of alpha-tocopherol for 180 days	Peripheral blood lymphocyte viability was restored, apoptosis was reduced, but changes in lymphocyte subsets did not differ significantly between groups, indicating that these changes were likely due to ART
**Not primarily antioxidant nutrients**					
Omega-3 fatty acids	Panchaud *et al*., 2006 [33]	Cystic fibrosis	17 (crossover)	A mixture of polyunsaturated fatty acids containing EPA, DHA, GLA, and stearic acid (dosage ranging from 57 to 1710 mg [body weight dependent])	LTB4/LTB5 ratio significantly decreased by 2.7-fold for the polyunsaturated fatty acid group, neutrophil membrane EPA level significantly increased by 3.2-fold, and the arachidonic acid/EPA ratio decreased in the polyunsaturated fatty acid group
	Okamoto *et al*., 2000 [34]	Asthma	Treatment: 7 Placebo: 7	10-20 g/day of perilla seed oil (rich in ALA) for 4 weeks	LTB4 and LTC4 significantly decreased, and peak expiratory flow, forced vital capacity, forced expiratory volume, and forced expiratory flow at 25% vital capacity significantly increased
	Okamoto *et al*., 2000 [35]	Asthma associated with lipometabolism	Treatment: 15 Placebo: 11	10-20 g/day of perilla seed oil for 4 weeks	In those with LTC4 suppression, LTC4 production significantly decreased with forced vital capacity, forced expiratory volume, and forced expiratory flow at 25% vital capacity values significantly increasing, and total cholesterol, LDL-C, and phospholipids significantly decreasing
	Barden *et al*., 2018 [36]	CKD	Treatment: 19 Placebo: 15	4 g/day of a combination of EPA and DHA for 8 weeks	LTB4 production did not change, LTB5 production significantly increased, plasma myeloperoxidase significantly decreased, and 18-HEPE, 14-HDHA, RvE1, RvE2, RvE3, and RvD5 significantly increased
	Zhao *et al*., 2007 [37]	Hypocholesterolemic subjects	23 (crossover)	ALA-rich diet for 6 weeks	Under LPS stimulation, PBMC production of IL-6, IL-1β, and TNF-α were significantly lower; IL-1β and TNF-α production decreased, and increase in serum ALA was also correlated with an increase in PBMC ALA and EPA, both of which were then inversely correlated with PBMC production of TNF-α
Vitamin B12	Chan *et al*., 2016 [38]	Pernicious anemia	Treatment: 30	1000 µg/day of Vitamin B12 (cyanocobalamin) intramuscularly until serum Vitamin B12 levels were within the normal range (200-900 pg/mL)	Leukocyte count significantly increased, lymphocyte count significantly decreased, absolute and relative CD8+ T cell counts increased significantly, leading to a nearly significant decrease in CD4/CD8 ratio, which was elevated at baseline, absolute CD3+ T cells and relative CD19+ cells increased significantly, complement 3 and IgG, IgA, and IgM increased significantly
Vitamin D3 (cholecalciferol)	Sotirchos *et al*., 2016 [39]	MS	10,400 IU: 19 800 IU: 21	10,400 IU/day (high dose) or 800 IU/day (low dose) of cholecalciferol for 6 months	Serum Vitamin D level significantly increased more in the high-dose group compared to the low-dose group, the proportion of Th17 cells and TEM CD4+ cells significantly decreased from baseline in the high dose group, only Th17 cells were significantly different between groups, and the reductions in Th17 cells and TEM CD4+ cells were strongly correlated
	Terrier *et al*., 2012 [40]	Vitamin D deficient lupus patients with low disease activity (SLEDAI no more than 8)	Treatment: 20	100,000 IU/week of cholecalciferol for 4 weeks and then 100,000 IU/month of cholecalciferol for 6 months with follow-up at 2 and 6 months	B and T cell homeostasis was successfully restored by increasing Tregs and decreasing Th1, Th17, and memory B cells
	Penna-Martinez *et al*., 2018 [41]	Addison’s disease	13 (crossover)	4000 IU/day of cholecalciferol for 3 months	Serum Vitamin D and its metabolite 1,25-dihydroxyvitamin D3 significantly increased, no patient remained deficient, resulting in the regulation of activated T cells and monocytes, with a decrease in expression of the MHC isotype that facilitates the autoimmune reaction on late-activated T cells
	Bartels *et al*., 2014 [42]	Crohn’s disease	Treatment: 10 Placebo: 10	1200 IU/day of cholecalciferol for 26 weeks	Serum Vitamin D significantly increased, CD80 expression on LPS-stimulated monocyte-derived dendritic cells (mo-DC) significantly decreased, mo-DC production of IL-10, IL-6, and IL-1β significantly decreased, shifting mo-DCs toward an anti-inflammatory state
	Walsh *et al*., 2010 [43]	Head and neck squamous cell carcinoma	Treatment: 16 Control: 16	Treated orally for three cycles (3 weeks total) with 4 µg/day of Vitamin D3 (1.25-D3) for 3 days followed by 4 days without treatment	Intratumoral CD4+ and CD8+ T cell counts significantly increased, infiltrating cells expressing CD69 (an activation marker) increased by approximately 10-fold, those who received Vitamin D3 had a significantly longer time to recurrence
	Kulbersh *et al*, 2009 [44]	Head and neck squamous cell carcinoma	Treatment: 11 Placebo: 6	No treatment or treated orally for three cycles (3 weeks total) with 4 µg/day of enteric Vitamin D3 (1.25-D3) for 3 days followed by 4 days without treatment	After 3 weeks, intratumoral CD34+ cells and DCs expressing immature markers significantly decreased, and the intratumoral mature DCs significantly increased in treatment group
	Lathers *et al*., 2004 [45]	Head and neck squamous cell carcinoma	Treatment: 6	20, 40, or 60 µg/day of Vitamin D3 (25-D3) for 6 weeks	Lower doses (20 and 40 µg) resulted in early temporary decreases in CD34+ cell count, but a higher dose (60 µg) led to a more enduring decrease; higher doses (40 and 60 µg) caused increases in MHC II expression, plasma IL-12 and IFN-γ, and T cell blastogenesis
	Hopkins *et al*., 2011 [46]	Colorectal cancer	Treatment: 23 Placebo: 23	800 IU/day of Vitamin D3 for 6 months	Serum Vitamin D significantly increased by 60% and CRP (32%), TNF-α (13%), IL-6 (32%), IL-1β (50%), IL-8 (15%), and an overall inflammation z-score (77%) significantly decreased, and changes in CRP and the overall inflammation score were more pronounced in men than women and non-NSAID users compared to NSAID users
	Neyestani *et al*., 2012 [47]	Type 2 diabetes	Treatment: 30 Placebo: 30	2 bottles/day of a yogurt drink, fortified with 500 IU of cholecalciferol for 12 weeks	CRP, IL-1β, IL-6, retinol-binding protein-4, and fibrinogen significantly decreased, and serum IgM and adiponectin significantly increased
	Meireles *et al*., 2016 [48]	Peritoneal dialysis and hemodialysis	Treatment: 20 Placebo: 18	50,000 IU of cholecalciferol twice/week for 12 weeks	Serum Vitamin D status, monocyte Vitamin D receptor, and CYP27B1 expression significantly increased, while CRP and IL-6 significantly decreased
	Burbank *et al*., 2017 [49]	Mild asthma	Treatment: 10	Three doses of 1200 mg gamma-tocopherol every 12 h	Plasma gamma-tocopherol and its metabolites were increased and were associated with a decrease in IL-8 production and likely reductions in airway neutrophil recruitment, and stimulated gene expression and production of other inflammatory cytokines and a gene for COX-2 decreased
	Burbank *et al*., 2018 [50]	Mild asthma	15 (crossover)	120 mg/day of gamma-tocopherol for 14 days	Asthmatic eosinophilia and inflammatory response to inhaled endotoxin were better managed with faster recovery from mucin hypersecretion
	Wiser *et al*., 2008 [51]	Moderate-to-persistent asthma in healthy subjects	Treatment: 8 Control: 8	Gamma-tocopherol was administered in escalating doses (one capsule 623 mg/day for 8 days, followed by an 8-day washout period, and then two capsules/day for 8 days)	g-CEHC increased with each dosing regimen and was significantly higher after two capsules compared to one capsule and ROS generation by PMA-stimulated PBMCs and serum 5-NITRO-GT significantly decreased after both dosages
Zinc	Bao *et al*., 2008 [52]	Sickle cell disease	Treatment: 18 Placebo: 18	25 mg zinc acetate 3 times/day for 3 months	Successfully increased erythropoiesis, decreased infection incidence, improved Th1 function, decreased soluble adhesion molecule concentrations, and decreased oxidative stress, while increasing antioxidant capacity
	Guo *et al*., 2013 [53]	Hemodialysis patients	Treatment: 40 Placebo: 25	11 mg/day of elemental zinc for 8 weeks	Plasma zinc concentration was restored, and the copper/zinc ratio was normalized, resulting in a decrease in oxidative stress, an increase in antioxidant activity, and normalization of T cell function
	Baum *et al*., 2010 [54]	HIV	Treatment: 115 Placebo: 116	Zinc (12 mg/day for females and 15 mg/day for males) for 18 months	Immunological failure was prevented (i.e., a drop in CD4+ T cell count below 200 cells/mm3)
	Meksawan *et al*., 2014 [55]	Type 2 diabetes with MetSyn	Treatment: 8 Placebo: 9	30 mg/day of elemental zinc for 8 weeks	Plasma TNF-α did not change, but transmembrane TNF-α expression on monocytes significantly increased
**Phytonutrients**					
*Aloe vera* polysaccharides	Lewis *et al*., 2013 [56]	Alzheimer’s disease	Treatment: 34	4 teaspoons/day of aloe polymannose multinutrient complex for 12 months	Increased CD14+ cells, decreased TNF-α and VEGF, decreased activity of B and T cells, and increased ratio of CD3+CD4+/CD3+CD8+ ratio correlated with increases in ASAS-cog and concentration scores
	McDaniel *et al*., 2020 [57]	Relapsing-remitting MS	Treatment: 15	Aloe polymannose multinutrient complex for 12 months	At 12 months, fewer total infections and decreased IL-1β and increased IL-2, TNF-α, EGF, and CD95+CD34+
Beta-glucans	Jesenak *et al*., 2013 [58]	Children with recurring respiratory tract infections	Treatment: 81 Placebo: 94	1 mL/5 kg/day of 10 mg of beta-glucans derived from mushrooms with 10 mg of Vitamin C/mL for 6 months	Increased all three Ig isotypes, increased NK cell count, and slower decline in the number CD8+ T cytotoxic lymphocytes, with a significant reduction in RTI incidence
	Demir *et al*., 2007 [59]	Newly diagnosed or relapsed metastatic breast cancer with lymphopenia	Treatment: 23 Placebo: 16	2 10 mg capsules/day for 14 days of baker’s yeast-derived beta-glucans	Increased proliferation and activation of peripheral blood monocytes with no adverse effects
	Ostadrahimi *et al*., 2014 [60]	Women with breast cancer	Treatment: 15 Placebo: 15	2 10 mg capsules of baker’s yeast-derived beta-glucans for 21 days	Milder decline of WBC, decreased serum IL-4, and increased IL-12 indicating a shift of the immune response toward Th1 cytokines and away from Th2 cytokines for a greater anti-tumor immune response
	Kirmaz *et al*., 2005 [61]	Allergic rhinitis	Treatment: 12 Placebo: 12	10 mg beta-glucans twice/day followed by a nasal provocation test using olive pollen and a sampling of nasal lavage fluid	Significant decrease in nasal lavage fluid containing IL-4 and IL-5 and a significant increase in IL-12, and decreased levels of eosinophils indicating beta-glucans’ ability to shift the immune system to a Th-1 mediated response
	Lee *et al*., 2016 [62]	ICU patients with pulmonary disease or trauma	Treatment: 8 Placebo: 7	Immune-modulating nutrients with 50 mg beta-glucans/200 kcal (derived from mushrooms) for 7 days	Significant increases in NKCA, slight increase in PBMC IL-12 release IL-12 concentration, and decrease in CRP
Bilberry	Karlsen *et al*., 2010 [63]	Subjects at-risk for CVD	Treatment: 31 Placebo: 31	330 mL/day of bilberry juice (diluted to 1 L using tap water) for 4 weeks	Significant decreases in plasma CRP, IL-6, IL-15, and monokine induced by IFN-γ, and increase in TNF-α
	Kolehmainen *et al*., 2012 [64]	Subjects with features of MetSyn	Treatment: 15 Placebo: 12	Bilberry-rich diet (equivalent to 400 fresh bilberries) for 8 weeks	Significant decreases in IL-6, IL-12, CRP, LPS, mRNA expression of C-C motif receptor 2, and monocyte differentiation-associated, and an overall decrease in low-grade inflammation
Black seed oil (Nigella sativa)	Gheita *et al*., 2012 [65]	Rheumatoid arthritis	40 (crossover)	500 mg black seed oil twice/day for 1 month	Significant decrease in WBC to within normal limits, an increase in blood glutathione, and self-reported decrease in morning stiffness and number of swollen joints
	Kalus *et al*., 2003 [66]	Allergic rhinitis	Treatment: 130 Placebo: 42	3 or 4 500 mg black seed oil capsules (depended on body weight) twice a day for 28 days	No significant changes in lymphocytes or lymphocyte subpopulations, but mean score of subjective symptom severity decreased
	Linnamaa *et al*., 2013 [67]	Pregnancy in women with atopic dermatitis and development of the condition in the infant	Treatment: 31 Placebo: 30	Black seed oil during 8^th^ to 16^th^ week of pregnancy, and for 3 months of breast feeding	Significantly lower IL-4 levels in breastmilk and significantly higher IFN-γ, thus Th1 response was enhanced Th2 response was suppressed in breast milk
Curcumin (turmeric)	Dolati *et al*., 2019 [68]	MS	Treatment: 25 Placebo: 25	80 mg of nanocurcumin daily for 6 months	Increases in Treg frequency, FoxP3 expression, and PBMC expression and secretion of TGF-β and IL-10
	Dolati *et al*., 2017 [69]	MS	Treatment: 50 Control: 35	80 mg of nanocurcumin daily for 6 months	Every microRNA expression significantly decreased in previously overexpressed T cells
	Kertia *et al*., 2012 [70]	Osteoarthritis	Treatment: 39 Placebo: 41	30 mg of curcuminoids 3 times/day	No significant difference between placebo and treatment group to reduce COX-2 secretion by synovial fluid monocytes
	Yang *et al*., 2015 [71]	Type 2 diabetes and type 2 diabetes with kidney disease	Treatment: 14	500 mg/day of curcumin for 15 days and an additional 30 days of treatment with type 2 diabetes with kidney disease	Plasma malondialdehyde and LPS decreased significantly, proteins associated with Nrf2 system function and IκB kinase significantly increased, and urinary albumin excretion considerably reduced
Frankincense (*Boswellia serrata*)	Sturner *et al*., 2018 [72]	MS	Intention-to-treat: 38 Per protocol: 28	2,400 mg/day for 8 months, patients also given option to extend treatment for up to 36 months	TGF-β, IL-4, and IL-5 significantly decreased during early and late treatment, IL-17A, IL-2, and IFN-γ significantly decreased only during late treatment, the number and volume of lesions decreased on magnetic resonance imaging, and reduced disease activity was maintained for up to 36 months for those who chose the extended treatment
	Baram *et al*., 2019 [73]	Ischemic stroke	Treatment: 41 Placebo: 39	Two 400 mg of boswellic acid three times/day for 30 days	Plasma TNF-α, PGE2, plasma MCP-1, IFN- γ inducible protein-10, and IL-8 significantly decreased, while the National Institutes of Health Stroke Scale score improved
Garlic	Mahdavi-Roshan *et al*., 2017 [74]	Coronary artery disease s/p angioplasty	Treatment: 21 Placebo: 21	Twice daily garlic tablets containing 1200 mg of allicin/tablet beginning 3 days after angioplasty	Flow mediated dilation in the brachial artery significantly improved, indicating better endothelial function, CRP significantly decreased after treatment, and it was negatively correlated with cholesterol efflux, which did not change at the end of the study
	Xu *et al*., 2018 [75]	Obese adults	Treatment: 24 Placebo: 24	Three 0.6 mg aged garlic extract capsules twice daily for 6 weeks	Percentage of NK T cells, IL-6, and TNF-α levels significantly lowered and percentage of γδ T cells significantly increased
	Ishikawa *et al*., 2006 [76]	Advanced colon, liver, or pancreas cancer	Treatment: 25 Placebo: 25	Four 500 mg aged garlic extract capsules for 3 months	Salivary cortisol and NKCA did not change
Ginger	Arablou *et al*., 2014 [77]	Type 2 diabetes	Treatment: 33 Placebo: 30	1,600 mg/day of ginger for 12 weeks	CRP, TNF-α, and PGE2 significantly decreased, and glycated hemoglobin, insulin, and insulin resistance, triglycerides, total cholesterol, and HDL-C:total cholesterol ratio improved
	Karimi *et al*., 2015 [78]	Obese women with breast neoplasms	Treatment: 10 Placebo: 10	Four 750 mg capsules of ginger for 6 weeks	CRP, IL-10, insulin, glucose, insulin resistance, LDL-C, and triglycerides decreased, and HDL-C and HDL-C/LDL-C increased
	Danwilai *et al*., 2017 [79]	Newly diagnosed cancer patients receiving moderate-to-high emetogenic chemotherapy	Treatment: 19 Placebo: 24	20 mg/day of ginger 3 days prior to chemotherapy until 64 days	SOD, catalase, glutathione peroxidase, and blood glutathione significantly increased, while MDA and nitric oxide significantly decreased
	Kulkarni *et al*., 2016 [80]	Category one tuberculosis	Treatment: 34 Placebo: 35	250 mg ginger extract twice/day for 30 days	TNF-α, MDA, and ferritin significantly decreased
	Mozaffari-Khosravi *et al*., 2016 [81]	Osteoarthritis	Treatment: 60 Placebo: 60	500 mg of ginger twice/day for 3 months	TNF-α and IL-1β significantly decreased
Hydrolyzed rice bran	Cholujova *et al*., 2013 [82]	Multiple myeloma	Treatment: 32 Placebo: 16	2 g/day of hydrolyzed rice bran for 3 months	NKCA, Th1 cytokines (i.e., IL-1β, IL-12, IL-17, and TNF-α), and Th2 cytokines (IL-5 and IL-6) significantly increased after 1 month, and peripheral myeloid and plasmacytoid DCs, IL-12, IL-17, IFN-γ, TNF-α, Th2 cytokines (IL-5 and IL-6)., IL-4, IL-6, IL-9, IL-10, and IL-13 significantly increased after 3 months
	Lewis *et al*., 2018 [83]	Non-alcoholic fatty liver disease	Treatment: 12 Placebo: 11	1 g/day of hydrolyzed rice bran for 90 days	Percent monocytes, percent eosinophils, IFN-γ, and IL-18 significantly increased, while ALP significantly decreased
	Lewis *et al*., 2018 [84]	HIV+ on stable ART	Treatment: 22 Placebo: 25	3 g/day of hydrolyzed rice bran for 6 months	Percentage change in CD8+ T cell count significantly decreased from baseline to 6 months and the CD4+/CD8+ ratio improved clinically in the hydrolyzed rice bran group to over 1.0 at 6 months
Isoflavones	Lesinski *et al*., 2015 [85]	Prostate cancer	32 (crossover)	Two slices/day of soy bread formulation containing 34 mg isoflavones/slice or one containing ground almonds	Th1 cytokines (IL-1β, TNF-α, and IFN-γ) and myeloid-derived suppressor cell-associated cytokines (IL-6, IL-13, IL-10, granulocyte colony-stimulating factor, granulocyte macrophage colony-stimulating factor, macrophage colony-stimulating factor, and VEGF), Tregs, and myeloid-derived suppressor cells decreased, and the percentage of CD56+ NK cells increased
	Ryu *et al*., 2016 [86]	Obese subjects with MetSyn	Treatment: 24 Placebo: 25	Six 2-g capsules/day of soybean leaf extract for 12 weeks	TNF-α, PAI-1, IL-6, insulin resistance, and free fatty acid level decreased
Mistletoe	Dohmen *et al*., 2004 [87]	Tumor patients	Treatment: 12	Subcutaneous treatment with aqueous mistletoe extract (15 ng mistletoe lectin/0.5 mL) twice/week for 48 weeks	NK cells significantly increased, while T lymphocytes (CD3+), cytotoxic T cells (CD8+), Th cells (CD3+CD4+), and T suppressor cells (CD3+CD8+) increased non-significantly
	Schink *et al*., 2007 [88]	Patients undergoing CRC resection	Treatment: 11 Placebo: 11	5 mg/mL mistletoe extra during administration of anesthesia	Postoperative NK cell suppression was avoided
	Enesel *et al*., 2005 [89]	Patients with various gastrointestinal cancers	Treatment: 40 Placebo: 30	Six vials/week of 60 mg/mL of aqueous mistletoe extract for 2 weeks preoperatively and 2 weeks postoperatively	Th cell (CD4+) count significantly increased in both day 1- and 2-weeks post-operation, T suppressor cell (CD8+) count significantly decreased at 2 weeks post-operation, IgA and IgM increased 2 weeks post-operation, and overall health status on the Karnofsky Performance Scale Index increased significantly
	Klopp *et al*., 2005 [90]	Larynx and pharynx carcinoma	Treatment: 10 Placebo: 10	Subcutaneous mistletoe extract Iscador® Qu Series 0 (ampoule with 1 ml), and Iscador® Qu 5 mg special (ampoule with 1 ml) for 12 weeks	Less decrease in ICAM-1, the number of adhering WBC on the derma near the tumor, and the number of migrating WBCs from the derma near the tumor
Resveratrol	Tome-Carneiro *et al*., 2013 [91]	Males with type 2 diabetes with hypertension	Treatment: 13 Placebo: 9	350 mg/day for the first 6 months and 700 mg/day for the subsequent 6 months of either conventional grape extract without resveratrol or grape extract with resveratrol	IL-6 significantly decreased only in the grape extract with resveratrol group, adiponectin and IL-10 significantly decreased, while the IL-6/IL-10 ratio increased, the transcript levels of several pro-inflammatory mediators (TNF-α, IL-8, and IL-1β) significantly downregulated in the grape extract with resveratrol group, the level of TNF-α in PBMCs was significantly positively correlated with mRNA levels of TNF-α, IL-1β, and NFKBIA gene in the grape extract with resveratrol group, and serum IL-6 was negatively correlated with adiponectin and PBMC expression of IL-1β
	Tome-Carneiro *et al*., 2013 [92]	Coronary artery disease	Treatment: 25 Placebo: 25	350 mg/day for the first 6 months and 700 mg/day for the subsequent 6 months of either conventional grape extract without resveratrol or grape extract with resveratrol	In the grape extract with resveratrol group, CRP non-significantly decreased in a dose-dependent manner at both 6 and 12 months, adiponectin increased at 12 months, and PAI-1 significantly decreased
Shiitake mushroom and its derivatives	Yoshino *et al*., 2000 [93]	Digestive cancers	Treatment: 28	2 mg intravenous lentinan 3 times every other day leading up to tumor resection surgery	Immune response shifted toward Th1 dominance, as shown by the increase in IFN-γ production and decreases in IL-4 and IL-6 production, and Th1 dominance was associated with higher survival time in those responders with unresectable tumors
	Wang *et al*., 2020 [94]	Non-small cell lung cancer	Treatment: 73 Control: 25	Intramuscular injection of 4 mg/day of lentinan in addition to chemotherapy for 12 weeks	CD3+CD56+ NK cells, CD3+CD8+ and CD3+CD4+ T cells, IFN-γ, TNF-α, and IL-12 significantly increased, and Treg induction, IL-10, and TGF-β1 significantly decreased along with the dominance from Th2 to Th1 response shifted
	Suknikhom *et al*., 2017 [95]	Peritoneal and epithelial ovarian cancer	Treatment: 14 Placebo: 14	Two capsules containing 500 mg of active hexose correlated compound 3 times/day for six cycles of chemotherapy	At the sixth cycle of chemotherapy, CD8+ T cell lymphocytes were significantly higher

## 3. Results

### 3.1. Nutrients

#### 3.1.1. Antioxidant nutrients

3.1.1.1. Acetyl-L-carnitine (ALCAR)

ALCAR is an antioxidant that has protective effects on mitochondrial function [2]. ALCAR has been found to facilitate mitochondrial fatty acid oxidation and enhance membrane phospholipid synthesis [3]. People living with HIV (PLWH) on highly active antiretroviral treatment (HAART) often have lipodystrophy (i.e., abnormal distribution of adipose and visceral fat) as a side effect, which may be partially mediated by mitochondrial damage [4]. Patients with HAART-related neurotoxicity also have depleted levels of ALCAR [5], giving it potential as an adjunct therapy.

PLWH with lipodystrophy were given 2 g/day of ALCAR for 48 weeks and showed an increase in mitochondrial DNA in circulating CD4+ T cells, implying an improvement in innate immunity and mitochondrial function [6]. However, mitochondrial DNA content in CD8+ T cells did not significantly change, which may be because CD8+ T cells are less sensitive to ALCAR. Therefore, ALCAR supplementation may have a protective effect on mitochondrial and immune function in PLWH on HAART with lipodystrophy.

3.1.1.2. Coenzyme Q10 (CoQ10) (ubiquinol)

CoQ10 is a mitochondrial enzyme that regulates oxidative phosphorylation [7] while acting as an antioxidant, mitigating reactive oxygen species (ROS) production, and pro-inflammatory signaling [8]. CoQ10 has been investigated in neurodegenerative disorders driven by excess inflammation, like multiple sclerosis (MS), a progressive neurodegenerative disease associated with increased infection susceptibility, reduced lifespan, and chronic dysregulated inflammation. MS is characterized by elevations of pro-inflammatory cytokines and decreases in anti-inflammatory molecules in the cerebrospinal fluid [9]. CoQ10 has also been found to suppress matrix metalloproteinases, which aid inflammatory cell infiltration into the central nervous system (CNS), also playing a large role in the pathogenesis of MS [96].

In one study, MS patients were randomized to receive either 500 mg/day of CoQ10 or placebo for 12 weeks [10]. After treatment, matrix metallopeptidase 9 (MMP-9) and pro-inflammatory cytokine (tumor necrosis factor [TNF]-a and interleukin [IL]-6) levels decreased significantly compared to the placebo group, while anti-inflammatory cytokine (IL-4 and transforming growth factor [TGF]-b) levels did not. Thus, CoQ10 not only reduced MMP-9 to alleviate CNS inflammation but it also lowered levels of inflammatory cytokines while maintaining levels of anti-inflammatory cytokines, making it quite useful as a therapy in MS.

Similarly, the ability of CoQ10 to reduce levels of inflammatory cytokines makes it a candidate to treat fibromyalgia, which is characterized by oxidative stress. The presence of ROS in fibromyalgia activates an inflammasome called NOD-like receptor family, pyrin domain containing 3 (NLRP3), which triggers the release of inflammatory cytokines, such as IL-1β and IL-18 [97].

In one study, fibromyalgia patients were randomized for 40 days to receive either 300 mg/day of CoQ10 (divided into three doses) or placebo to measure changes in NLRP3 gene expression and serum cytokine levels [11]. After treatment, NLRP3 and IL-1β gene expressions were downregulated, with concurrent decreases in serum IL-1β and IL-18 levels in the CoQ10 group compared to placebo. Only half of the treatment group participants experienced a small reduction in symptoms. Therefore, additional therapies are likely needed to enhance improvements. Regardless of these findings, CoQ10 shows promise as an anti-inflammatory supplement in fibromyalgia.

Another condition driven by inflammation and the expression of prothrombotic factors is antiphospholipid syndrome. Antiphospholipid syndrome is a hypercoagulable condition that is characterized by thrombosis development in the veins and arteries and endothelial dysfunction [98]. These pathologies lead to an increase in the influx of low-density lipoproteins (LDL) into macrophages, promoting monocyte activation, and atherosclerosis [99]. CoQ10 has been found to mitigate the oxidative stress in antiphospholipid syndrome leukocytes *in vitro*, prompting a parallel *in vivo* study. In this study, antiphospholipid syndrome patients were randomized for 1 month to either receive 200 mg/day reduced CoQ10 or placebo [12]. After treatment, IL-6, IL-8, vascular endothelial growth factor (VEGF), macrophage inflammatory protein (MIP)-1α, IL-1β, and TNF-α levels in monocytes and oxidized LDL level decreased. Endothelial function also improved, according to a significant reduction in plasma vascular cell adhesion protein 1 (VCAM-1) and a modulation of nitric oxide, which were overexpressed before treatment. Therefore, CoQ10 appears to be effective in decreasing inflammation and severity of thrombosis in antiphospholipid syndrome.

3.1.1.3. Lipoic acid

Lipoic acid is an antioxidant that regulates mechanisms of inflammation in several different chronic diseases. For example, it has been found to enhance Th cell function [100], mitigate ROS production [101], and increase glutathione availability [102]. Glutathione plays a key role in T cell activation, IL-2-dependent proliferation, and overall antibody-mediated cytotoxicity and is depleted in conditions like HIV. Thus, lipoic acid may be promising as a therapeutic candidate for PLWH. HIV-infected men and women, who were non-responsive to HAART, were randomized to either 300 mg α-lipoic acid 3 times daily or placebo for 6 months and had an increased blood glutathione level [13]. The α-lipoic acid arm also saw an enhanced lymphocyte proliferative response compared to the placebo group. These results imply that the recovery of the lymphocyte response may have been due to the increase in glutathione. Because glutathione deficiency and impaired lymphocyte function are characteristic of HIV, these results suggest that α-lipoic acid could be a potential HIV adjunct therapy. This study did not demonstrate any changes in CD4+ and CD8+ T cell counts, perhaps due to the HAART non-responsiveness within the study population, warranting further investigation in a treatment-responsive group.

Lipoic acid also shows promise in conditions such as metabolic syndrome (MetSyn), which is characterized by systemic inflammation and endothelial dysfunction. Patients with MetSyn were randomized for 4 weeks to receive either 300 mg/day of α-lipoic acid or placebo [14]. Those taking α-lipoic acid saw a 15% reduction of IL-6 and a 14% reduction of plasminogen activator inhibitor (PAI)-1, significantly different from placebo. These markers contribute to atherosclerosis, which is common in MetSyn [103]. Therefore, α-lipoic acid may be a useful therapy to mitigate endothelial dysfunction and MetSyn severity.

MS occurs when T cells travel across the blood-brain barrier into the CNS [104], causing autoreactivity, neuroinflammation, and demyelination [105]. The migration of T cells into the CNS relies on binding with ligands such as intercellular adhesion molecule (ICAM)-1 on endothelial cells [106]. Thus, an increased concentration of circulating ICAM-1 is often associated with inflammatory diseases [107,108], atherosclerosis [109], and the brain lesions that occur with MS [110]. T cell migration is further facilitated by the production of MMP-9, which breaks down extracellular matrix proteins to allow entry into the CNS [111]. Thus, lipoic acid may be useful in counteracting the various inflammatory cascades so prevalent in MS.

In one study, MS patients were randomly assigned for 14 days to either placebo twice/day or one of three different doses of lipoic acid: 600 mg twice/day, 1200 mg in the morning and placebo in the evening, or 1200 mg twice/day [15]. Subjects in the two 1200 mg lipoic acid groups had significantly higher serum lipoic acid levels than those in the 600 mg group. Serum lipoic acid levels were inversely correlated with the mean change in serum MMP-9 and ICAM-1 levels in a dose-dependent manner for the latter but not the former. Therefore, it can be concluded that lipoic acid has potential as an adjunct therapy for MS, as it reduces MMP-9 and ICAM-1 activity, thus mitigating T cell entry into the CNS. Furthermore, a higher dose appears to be more effective while remaining well-tolerated.

3.1.1.4. N-acetyl cysteine

N-acetyl cysteine (NAC) is a precursor to glutathione, which has a wide variety of purposes, including defense against oxidative stress [112], regulation of metabolic function [113], and regulation of T cell and NK cell function. Thus, low levels of glutathione can be physiologically detrimental by impairing cytotoxic T cell activity and natural killer cell activity (NKCA) [114,115], cytokine production and response [116], and Th1 responses [117]. Therefore, glutathione deficiency may play a large role in the pathology of many chronic diseases, such as HIV [118], neurodegenerative disorders [119], hepatorenal syndrome [120], and many more. HIV is characterized by significant immunosuppression, primarily decreases in CD4+ T cell count [121]. Studies reveal that HIV may increase cysteine catabolism, which depletes cysteine and glutathione [122], both of which can generally regulate and inhibit nuclear factor-Kappa B (NF-κB), reducing viral replication [123]. Thus, NAC may hold promise as a therapy for HIV by restoring glutathione levels, therefore reducing viral burden.

PLWH were randomized for 8 weeks to receive 8000 mg/day of NAC or a placebo [16]. Whole blood glutathione and glutathione produced by T cells in the NAC group significantly increased with no change in the placebo group. NAC supplementation was also associated with improved 2-3 years of survival and minimal side effects. Similar results were demonstrated in two studies using NAC in PLWH with and without antiretroviral therapy (ART) [17]. In each trial, patients were randomized for 7 months to receive either individually adjusted doses of NAC (depending on initial plasma glutamine levels ranging from 0.6 g to 3.6 g/day) or a placebo. At baseline, NKCA, stimulation indices, plasma glutamine, and serum albumin were low compared to HIV- subjects, regardless of whether subjects received ART or HAART (with at least one protease inhibitor). In both studies, NAC treatment yielded a significant increase in NKCA to almost normal levels. In study one, the placebo group demonstrated ART failure with a significant increase in viral load; in contrast, the viral load did not increase in the NAC treated group. However, it should be noted that the NAC group had a higher baseline viral load. In study two, NAC had no significant effect on viral load. In study one, NAC also significantly increased plasma glutamine and albumin levels and decreased IL-6. The effects were not significant in study two, but the effects on albumin and IL-6 were significant when the data from both studies were combined. Overall, NAC appears successful in restoring HIV patients’ immune function, regardless of whether they received ART or HAART.

In another study in HIV, patients were randomized to receive either 600 mg/day NAC or a placebo in addition to ART, and outcomes were measured every 60 days for 180 days [17]. Those in the NAC group saw a significant increase in CD4+ T cell count at 60 days, whereas the control group did not see an increase until 120 days. These changes were at least partially due to the significant decreases in viral load (i.e., virus replication) in both groups. While the decrease in viral load was attributed to ART, it was amplified by NAC. Overall, the data from these HIV studies suggest that NAC is an effective way to restore immunocompetence in HIV.

NAC has also proven to be beneficial in several other chronic conditions. Lupus is characterized by a depleted glutathione level, which regulates mammalian target of rapamycin (mTOR) in lupus T cells [124]. mTOR inhibits the development of CD4+CD25+forkhead box P3(FoxP3)+ regulatory T cells (Tregs) [125], which are deficient in lupus patients [126]. Using NAC to inhibit mTOR has improved lupus outcomes [127]. In one study, lupus patients were randomized for 3 months to receive one of three doses of NAC (1.2, 2.4, or 4.8 g/day) or placebo [18]. At baseline, the peripheral blood lymphocyte glutathione level was reduced compared to healthy controls, but not in whole blood, suggesting that dysfunction is limited to the immune system. With NAC, peripheral blood lymphocyte and whole blood glutathione increased after 1 and 2 months. NAC treatment also significantly increased mitochondrial mass and spontaneous apoptosis rate in CD4-/CD8- double-negative T cells. At baseline, mTOR activity was significantly elevated, but decreased significantly after 2 and 3 months of NAC treatment, and the effect was reversed after 1 month without treatment. At baseline, FoxP3+ cells were reduced in the CD4+/CD25+ compartment compared to controls, but after treatment the percentage of these cells was significantly increased in all patients. None of these effects was seen in the placebo group. The Systemic Lupus Erythematosus Disease Activity Index (SLEDAI) improved significantly in the NAC group compared to placebo. NAC was well-tolerated in all patients taking up to 2.4 g/day, while reversible nausea occurred in about one-third of the patients taking 4.8 g/day. Thus, up to 2.4 g/day, NAC supplementation appears to be an effective therapeutic approach to lupus through increases in glutathione and subsequent inhibition of mTOR and restoration of double-negative T cell function and Treg count.

Cystic fibrosis is another chronic condition with glutathione deficiency, characterized by increased recruitment of neutrophils to the airway [128], partly due to excessive IL-8 production by airway epithelial cells [129]. These neutrophils then release effectors, such as elastase, which contribute to airway inflammation [130]. Neutrophil abnormality is further exacerbated by glutathione deficiency, due to depressed activity of antioxidant enzymes [131]. Thus, NAC shows promise as a therapy to reduce airway inflammation in cystic fibrosis.

In one study, pediatric cystic fibrosis patients were randomized for 4 weeks to one of three doses of NAC: 0.6, 0.8, or 1.0 g 3 times/day [19]. At baseline, the glutathione level in cystic fibrosis patients was significantly lower compared to healthy controls. NAC treatment significantly increased both whole blood and neutrophil levels of glutathione in the cystic fibrosis patients with no significant differences between dosages. NAC treatment also significantly reduced airway neutrophil count, especially in those with baseline values outside of the normal range. Sputum IL-8 level was also significantly reduced with NAC treatment. Furthermore, the number of elastase-releasing neutrophils in the airway and overall sputum elastase activity, which is considered the best predictor of pulmonary function in cystic fibrosis, was significantly decreased after treatment [130]. Therefore, high-dose NAC supplementation effectively augments the glutathione level in cystic fibrosis and mitigates airway neutrophil count and release of inflammatory effectors.

In addition, NAC has therapeutic potential in the field of nephrology, as it has been found to reduce oxidative stress and production of advanced glycation end products in patients with chronic kidney disease (CKD) undergoing peritoneal dialysis [132]. Several inflammatory markers are associated with CKD, such as C-reactive protein (CRP), IL-6, ICAM-1, and TNF-α [133]. Susceptibility to infection in CKD can also be increased with a deficiency of complement component 3, as it is an important protein in the complement system, which defends against pathogens [134]. In one study, CKD patients receiving continuous ambulatory peritoneal dialysis were randomized for 8 weeks to receive either 1.2 g/day of NAC or placebo [20]. The NAC group saw significant decreases in procalcitonin, IL-6, IL-1, and complement component 3 compared to the control group. These improvements in inflammation and endothelial dysfunction likely mitigate the risk of cardiovascular disease (CVD), the leading cause of mortality in CKD [135]. Thus, NAC appears effective in reducing inflammation and cardiovascular comorbidities in CKD patients receiving continuous ambulatory peritoneal dialysis.

NAC supplementation has also been investigated in acute conditions. For example, toxicity occurs in lead exposure because it can inactivate glutathione and interfere with antioxidant activity, such as superoxide dismutase (SOD), catalase, and glutathione peroxidase [136]. These enzymes utilize hydrogen peroxide [137] and are therefore significantly elevated in cases of oxidative stress such as lead exposure [138]. Thus, lead-exposed workers were randomized for 12 weeks to receive 200, 400, or 800 mg/day of NAC or no treatment [21]. After treatment, blood lead concentration significantly decreased in all treatment groups with no change in the control group. Erythrocyte SOD activity significantly decreased in the 200 and 400 mg groups compared to baseline and in the 800 mg group compared to both baseline and control. Leukocyte SOD activity significantly decreased in the 200 mg group compared to control. Leukocyte catalase activity significantly decreased in all treatment groups compared to baseline. Erythrocyte glutathione peroxidase activity significantly decreased in the 200 and 800 mg groups compared to both baseline and control. Leukocyte malondialdehyde (MDA), a marker of lipid peroxidation, significantly decreased in the 200 mg group (compared to baseline), serum MDA level in the 400 mg group (compared to both baseline and control), and both serum and leukocyte MDA levels in the 800 mg group (compared to control), indicating a decrease in oxidative stress in all groups. The largest decline occurred in the 800 mg group, and the dose of NAC was significantly correlated with the reduction in oxidative stress. Correlations were also found between MDA level and change in glutathione peroxidase activity in erythrocytes and change in SOD activity in leukocytes. Therefore, NAC successfully decreased blood lead content and oxidative stress in a dose-dependent manner, as evidenced by reductions in MDA and concurrent normalization of the activity of several antioxidant enzymes.

Burn-induced edema is another acute condition involving oxidative stress and inflammation [139]. Burn injuries are associated with increased pulmonary lipid peroxidation [140]. However, NAC was successful in decreasing bacterial translocation and immunosuppression after burns by elevating glutathione and decreasing the MDA level [141]. Leukocyte cell surface markers, such as CD11a, CD18, and CD97, contribute to inflammation after a burn through endothelial leukocyte activation and adhesion to the endothelium and can be used to measure intensity of inflammation after thermal injury [142]. Thus, severe burn patients were randomly assigned for 6 days to receive either NAC (150 mg/kg bolus followed by continuous administration of 12 mg/kg/h) or placebo [22]. Compared to the placebo group, the NAC group had significantly lower levels of granulocyte CD11a and CD18 on days 4-6, granulocyte CD97 on days 2-6, and granulocyte CD49d on day 2. Compared to the placebo group, the NAC group had significantly lower levels of lymphocyte CD11a and CD49d on days 3-6. Compared to the placebo group, the NAC group had significantly lower levels of monocyte CD49d on days 4-6 and monocyte CD97 on days 3-6. The procalcitonin level was significantly lower in the NAC group than the placebo group on days 2-4. The NAC group required significantly less inotropic and vasopressor drugs than the placebo group on days 4-6, perhaps by reducing edema and subsequent increased fluid requirements. Therefore, NAC supplementation successfully decreased inflammation during the acute phase of thermal injury, as evidenced by a decrease in the expression of leukocyte cell surface markers and mitigation of hypovolemia.

3.1.1.5. Selenium

Selenium is an important micronutrient, but an inadequate intake of dietary selenium is common [143], leading to diminished humoral, and cell-mediated immune responses [144]. Selenium deficiency is associated with the occurrence and progression of certain viral infections, e.g., HIV [145], and increased mortality risk in infected individuals [146]. However, selenium supplementation has been found to increase NKCA, T cell proliferation, and vaccine-induced immunity [144].

In one study, PLWH were randomized for 9 months to receive either 200 μg/day of high-selenium yeast or placebo [23]. At baseline, the mean pre-treatment selenium level was slightly decreased, but still comparable to healthy controls. At 9 months follow-up, serum selenium concentration significantly increased in the selenium group, but not in the placebo group. An increase in serum selenium concentration was significantly negatively associated with the change in viral load, which was negatively associated with CD4+ T cell count, when controlling for the pre-treatment value. In other words, it appears that the increase in CD4+ T cell count was not a direct result of the treatment, but rather a result of the decrease in viral load. Follow-up analysis divided the selenium group by treatment effect. Responders were defined as having a mean change in serum selenium more than 3 standard deviations above that of the placebo group (above an increase in 26.1 μg/L). The responders showed significantly greater treatment adherence, less of an increase in viral load, and a greater increase in CD4+ T cell count compared to both the non-responders and placebo. Therefore, to yield significant benefits from selenium supplementation in PLWH, the serum selenium concentration must be elevated. Nonetheless, selenium intake appears to suppress the viral burden of HIV-1, allowing for improvements in CD4+ T cell count. In another study in PLWH, ART-naive patients were randomized for 24 months to receive either 200 μg/day of selenium or placebo [24]. Those who received selenium had a significantly reduced rate of CD4+ T cell depletion (43.8%) compared to those who received placebo. In terms of viral suppression, the odds ratio was 1.18 in favor of selenium. However, this was not significant enough to conclude a treatment effect on viral burden.

Selenium supplementation has also been found to be beneficial in several other chronic conditions. In one study, type 2 diabetes mellitus (T2DM) patients were randomized for 3 months to receive either 960 μg/day of selenium or placebo, and a group of non-diabetic subjects served as a comparison group [25]. At baseline, T2DM patients had significantly higher (80%) peripheral blood mononuclear cell (PBMC) NF-κB binding activity compared to non-diabetic controls. NF-κB regulates adhesion molecules and genes involved in vascular remodeling and formation of atherosclerotic plaques [147]. Thus, activation of NF-κB is linked with microvascular complications in diabetes, for example, retinopathy and nephropathy [148]. Most of the T2DM patients in the cohort suffered from retinopathy, which may account for the elevated NF-κB activity at baseline. In addition, an enzyme called selenium-dependent glutathione peroxidase can decrease peroxide formation, which is one of the many inflammatory stimuli for NF-κB activation [149]. Thus, increases in selenium-dependent glutathione peroxidase activity would lead to decreases in NF-κB activity. Indeed, plasma selenium and activity of selenium-dependent glutathione peroxidase were significantly increased and NF-κB activity was significantly decreased in the selenium group. In fact, NF-κB activity reached a level like that of the non-diabetic group, whereas no change occurred in the placebo group. Therefore, the significant decrease in NF-κB activity and increase in selenium-dependent glutathione peroxidase with selenium supplementation may represent a decrease in oxidative stress and a subsequent alleviation of diabetic retinopathy.

Faulty glutathione peroxidase activity also results in lack of mycobacterium clearance, increasing risk of tuberculosis (TB) in those who are selenium deficient. TB patients, therefore, have increased production of ROS [150] and high rates of lipid peroxidation [151]. Therefore, selenium shows promise in restoring glutathione peroxidase function and treating TB. In one study, pulmonary TB patients were randomized for 6-8 months to receive either 100 μg/day of selenium or placebo in addition to an anti-TB drug [26]. Both groups had an elevated leukocyte count at baseline, but after treatment, the selenium group had a lower count than the controls. Immunoglobulin (Ig)G and IgM significantly increased in the treatment group, compared to the control group, and CD4+ and CD8+ T cells significantly increased as well. MDA was significantly lower, while SOD, catalase, glutathione, and total antioxidants were higher in the treatment group compared to the control group. The increases in immunoglobulins and T cells indicate increased immunity in response to antibiotics and enhanced recovery. In addition, the decrease in MDA indicates a decrease in lipid peroxidation, likely because of increased glutathione peroxidase activity in addition to increased activity of antioxidant enzymes. Overall, selenium supplementation appears to decrease oxidative stress and increase immune function in TB patients.

Asthma is another condition in which selenium deficiency can impair glutathione peroxidase function and increase infection susceptibility. Asthma is characterized by infiltration and activation of immune cells into the airway [152], which release inflammatory mediators and increase their expression of adhesion molecules [153]. Studies have reported increased concentrations of ICAM-1, vascular cell adhesion molecule (VCAM)-1 and E-selectin in the plasma of asthmatic subjects [154], which can potentially be mitigated with selenium supplementation. Corticoid-dependent asthmatics were randomized for 6 months to receive either 200 mg/day selenium or no selenium, and a group of healthy controls was used for comparison [27]. All asthmatics had a suboptimal selenium concentration at baseline. After selenium treatment, PBMC expression of CD11a, CD11b, and CD62L significantly decreased. In human umbilical vein endothelial cells (HUVECs), those who did not receive selenium had significant increases in the expression of VCAM-1, P-selectin, and E-selectin expression compared to controls. In contrast, those who received selenium had significant decreases in human umbilical vein endothelial cell expression of ICAM-1, VCAM-1, P-selectin, and E-selectin. Therefore, this study demonstrates the increased expression of adhesion molecules in asthmatic individuals and shows that selenium supplementation decreases their expression on both PBMCs and endothelial cells.

3.1.1.6. Vitamin C (ascorbate)

Vitamin C is widely lauded for its antioxidant properties, and thus it may be of use in disease states characterized by chronic inflammation and elevated ROS. Patients with end-stage renal disease or hyperlipidemia have atherogenic LDL that can contribute to vascular dysfunction and atherosclerosis [155]. These patients often undergo hemodialysis to remove LDL from the blood, which can prevent cardiac events [156]. However, the treatment is associated with increased ROS (e.g., hydrogen peroxide) by polymorphonuclear neutrophils [157], causing oxidative stress and subsequent lipid oxidation [158]. Thus, antioxidants such as Vitamin C may be potential preventative therapy for atherosclerosis in those patients undergoing hemodialysis.

Hyperlipidemic and uremic patients who regularly received hemodialysis were administered 2.5 g Vitamin C in 150 mL of 5% albumin solution during their hemodialysis session [28]. At baseline, the plasma hydrogen peroxide level in these patients was elevated compared to healthy controls. The plasma hydrogen peroxide level was further elevated by 60-70% after hemodialysis, indicating an increase in oxidative stress. However, after Vitamin C administration, hydrogen peroxide was reduced by 80-93% compared to no supplementation. The degree of reduction was like that in patients on lipid-lowering medications, such as simvastatin. The percentage of intracellular ROS production of polymorphonuclear neutrophils was significantly lower in those taking Vitamin C supplementation. In addition, Vitamin C treatment suppressed monocyte chemoattractant protein (MCP)-1 and TNF-α production, which is not accomplished in hemodialysis alone. Finally, while hemodialysis normally decreases LDL lipid oxidation markers, such as phosphatidylcholine hydroperoxide and MDA, these markers were further reduced with Vitamin C supplementation. When investigating the long-term (6-month) effects of the treatment, it was discovered that biweekly hemodialysis with Vitamin C supplementation significantly decreased CRP more than in those receiving hemodialysis alone. Therefore, not only does Vitamin C augment the benefits of hemodialysis but it also mitigates the detriments through inflammatory and oxidative stress pathways, both in the short- and long-term.

3.1.1.7. Vitamin E (d-alpha- and gamma-tocopherol)

Vitamin E is often supplemented as one of two isoforms: Alpha-tocopherol or gamma-tocopherol. Alpha-tocopherol supplementation has been shown to decrease LDL oxidation and platelet aggregation, improve endothelial function, and decrease monocyte proatherogenic activity, decreasing the overall risk of atherosclerosis, and other vascular complications [159]. Activated monocytes in T2DM patients produce more ROS [160], and hyperglycemia causes an increase in monocyte-endothelium adhesion [161], making them more prone to atherogenesis [162]. Therefore, alpha-tocopherol is a good candidate for those with T2DM.

In one study of T2DM patients with and without macrovascular disease and a healthy control group for comparison, all subjects were given 1200 IU/day of alpha-tocopherol for 3 months [29]. At baseline, both T2DM groups trended toward increased LDL oxidative susceptibility, had significantly increased monocyte superoxide anion, TNF-α, and IL-1β secretion, and had significantly greater monocyte-endothelium adhesion and level of sICAM-1, compared to control. These levels were insignificantly more elevated in the macrovascular disease group. After treatment, the lag phase of LDL oxidation significantly increased in all three groups. Supplementation also caused a significant decrease in superoxide anion, IL-1β, and TNF-α release (with greater inhibition of IL-1β than TNF-α), a significant decrease in monocyte-endothelium adhesion in HUVECs, and a significant decrease in sCAMs compared to baseline for all three groups. Therefore, alpha-tocopherol supplementation reversed the baseline elevations in pro-atherogenic and pro-inflammatory monocyte activity in T2DM patients with and without macrovascular complications.

In another trial of T2DM patients using the same study design by the previous scientists [30], those subjects with macrovascular disease had elevated CRP compared to those subjects without macrovascular disease and controls. In addition, both T2DM groups had elevated monocyte IL-6 production compared to the controls. After treatment, CRP and monocyte IL-6 production significantly decreased in all three groups compared to baseline. Thus, these results further support the use of alpha-tocopherol as a supplement to decrease inflammation and risk of atherosclerosis in individuals with T2DM.

The efficacy of alpha-tocopherol in protecting against LDL oxidation is also clinically important in hypertriglyceridemic individuals, who also exhibit higher ROS production by polymorphonuclear cells (PMNs) [163]. In one study, normotriglyceridemic and hypertriglyceridemic individuals were enrolled for 6 weeks to receive 600 IU/day of alpha-tocopherol [31]. After alpha-tocopherol treatment, the phorbol 12-myristate 13-acetate (PMA)-stimulated chemiluminescence of PMNs and whole blood (measured by changes in heights of peaks and plateau levels) significantly decreased in both groups, indicating an overall decrease of superoxide anion production. However, the chemiluminescence of PMNs in response to stimulation with LDLox (LDL incubated with copper(II) sulfate) significantly increased compared to baseline in both groups. After treatment, lipopolysaccharide (LPS)-stimulated PBMC production of TNF-α, IL-1β, and IL-8 significantly decreased in both groups compared to baseline. However, stimulation with LDLox and naive LDL did not induce cytokine production. Therefore, alpha-tocopherol supplementation appears to have differential inflammatory effects depending on the stimulus but has potential in decreasing PMA-stimulated superoxide anion production and LPS-stimulated cytokine release in both normotriglyceridemic and hypertriglyceridemic individuals.

In states of chronic oxidative stress, alpha-tocopherol has been found to protect the viability of peripheral blood lymphocytes [164], giving it potential as a therapy for HIV, characterized by a decline in these lymphocytes [165]. In one study, PLWH on ART were randomized for 180 days to receive 800 mg/day of alpha-tocopherol or placebo [32]. After treatment, the percentage of viable lymphocytes significantly increased in both groups, which negatively correlated with the percentage of lymphocytes in apoptosis. The reduction in lymphocyte apoptosis was significantly greater in the treatment group compared to the control group. The plasma level of HIV-1 RNA significantly decreased in both groups after 120 days of treatment but was not different between groups. CD4+ T cell count significantly increased after 60 days in both groups with no difference between groups. CD8+ T cell count did not significantly change, therefore showing an increase in CD4/CD8 ratio due to ART, with no difference between groups. Therefore, alpha-tocopherol supplementation successfully restored peripheral blood lymphocyte viability and reduced apoptosis. However, changes in lymphocyte subsets did not differ significantly between groups, indicating that these changes were likely due to ART.

The other isoform of Vitamin E, gamma-tocopherol, has been found to exhibit effects that alpha-tocopherol does not, such as cyclooxygenase (COX)-2 and lipoxygenase inhibition [166], reactive nitrogen species trapping [167], and inhibition of cytokine production, for example, IL-8, which contributes greatly to airway inflammation by acting as a chemoattractant for neutrophils [51]. Gamma-tocopherol has also been found to inhibit IL-1β-stimulated epithelial cells, which are initially affected by ozone [168]. Furthermore, studies have found a negative correlation between plasma gamma-tocopherol level and lung function in those with airway inflammation [168], giving it potential in treating asthma.

In one study, individuals with mild asthma were given three doses of 1200 mg gamma-tocopherol every 12 h [49]. By 30 h, plasma gamma-tocopherol and its metabolite 2, 7, 8-trimethyl-2-(b-carboxyethyl)-6-hydroxychroman (g-CEHC) significantly increased, but plasma alpha-tocopherol decreased. LPS-stimulated IL-1β and IL-6 PBMC production significantly decreased compared to baseline. LPS-stimulated IL-8 production did not change, but plasma g-CEHC and change in IL-8 production were negatively correlated. On PBMC gene evaluation, IL-6, IL-1β, and PTGS2 (which encodes COX-2) were most impacted by gamma-tocopherol supplementation, with significant reductions in LPS-stimulated expression of IL-6 and slight reductions in IL-1β and PTGS2 expression. Therefore, short-term gamma-tocopherol supplementation increased plasma gamma-tocopherol and its metabolites, which was associated with a decrease in IL-8 production and likely reductions in airway neutrophil recruitment. In addition, gamma-tocopherol supplementation decreased stimulated gene expression and production of other inflammatory cytokines and a gene for COX-2, further supporting its inhibitive abilities.

In another study of mild asthmatics, subjects were randomized for 14 days to receive either 120 mg/day of gamma-tocopherol or placebo [50]. After treatment, serum gamma-tocopherol and g-CEHC significantly increased, and plasma alpha-tocopherol decreased in the treatment group, but not in the control group. Gamma-tocopherol treatment also resulted in a significant decrease in the percentage of sputum eosinophils, eosinophils/mg of sputum, mucin 5AC (which has specifically been found in abundance in mucous plugs from fatal asthma cases [169]), and total mucins compared to placebo. Inhaled LPS stimulation significantly increased the percentage of sputum neutrophils and neutrophils/mg of sputum in both groups 6 h after stimulation, but the increase in the placebo group was significantly greater than that of the gamma-tocopherol group. Linear mixed modeling showed that gamma-tocopherol treatment significantly decreased the percentage of sputum neutrophils at 6 and 24 h after LPS stimulation. Inhaled LPS stimulation significantly increased mucin 5AC content in both groups six h after stimulation, but total sputum mucin decreased in both groups compared to baseline. Linear mixed modeling found that the total sputum mucin present was significantly less in the treatment group compared to control. Mucociliary clearance was also slowed following LPS stimulation in the placebo group compared to post-intervention, whereas this did not occur in the gamma-tocopherol group. Therefore, gamma-tocopherol supplementation overcame asthmatic eosinophilia and inflammatory response to inhaled endotoxin with faster recovery from mucin hypersecretion.

In another study of both healthy individuals and those with moderate to severe persistent asthma, gamma-tocopherol was administered in escalating doses (one capsule 623 mg/day for 8 days, followed by an 8-day washout period, and then two capsules/day for 8 days) [51]. The level of g-CEHC increased with each dosing regimen and was significantly higher after two capsules compared to one capsule with no difference between healthy and asthmatic individuals. The washout period level fell to the baseline level. ROS generation by PMA-stimulated PBMCs and serum 5-NITRO-GT, which is generated through nitration of reactive nitrogen species [170], significantly decreased after both dosages. Therefore, systemic oxidative and nitrosative stress appeared to decrease. With gamma-tocopherol treatment, LPS-stimulated PBMC production of IL-1β, IL-6, TNF-α, MCP-1, and macrophage inflammatory protein (MIP)-1α significantly decreased with no difference between healthy and asthmatic individuals. Therefore, gamma-tocopherol supplementation appears successful in mitigating an inflammatory response to an inhaled endotoxic challenge, proving its usefulness in asthma.

#### 3.1.2. Not primarily antioxidant nutrients

3.1.2.1. Omega-3 fatty acids

Essential omega-3 fatty acids (EΩ3FA), such as docosahexaenoic acid (DHA), eicosapentaenoic acid (EPA), and a-linolenic acid (ALA), are necessary for human health. These fatty acids must be obtained from the diet because the human body is limited in its ability to synthesize them. EΩ3FA are found in various foods such as fish, nuts, and seeds [171]. EPA and DHA have been found to modulate inflammation, often through competitive inhibition of arachidonic acid use by COX and lipoxygenase enzymes [172], producing eicosanoids that are less pro-inflammatory. One example is leukotriene B5 (LTB5), which is significantly less active than its arachidonic acid-derived counterpart, LTB4 [173]. Thus, supplementation with EΩ3FA has potential in many inflammatory conditions characterized by a high LTB4/LTB5 ratio.

In one crossover study, cystic fibrosis patients were randomized for 6 months to receive either a mixture of polyunsaturated fatty acids containing EPA, DHA, γ-linolenic acid (GLA), and stearic acid (dosage ranging from 57-1,710 mg depending on body weight) or placebo [33]. Cystic fibrosis is characterized by bronchial airway inflammation, often due to neutrophil chemotaxis in response to IL-8 and LTB4 [174]. After treatment, the LTB4/LTB5 ratio significantly decreased by 2.7-fold for the polyunsaturated fatty acid group with no change in the placebo group. Neutrophil membrane EPA level significantly increased by 3.2-fold and the arachidonic acid/EPA ratio decreased in the polyunsaturated fatty acid group. Neutrophil membrane dihomo-γ-linolenic acid level increased non-significantly in the polyunsaturated fatty acid group. Dihomo-γ-linolenic acid is a metabolite of GLA, which inhibits arachidonic acid metabolism, decreasing LTB4 synthesis [175]. Indeed, this change was accompanied by a nearly significant decrease in arachidonic acid level. Thus, the increase in dihomo-γ-linolenic acid level was likely a result of GLA consumption and contributed to the modulation of the LTB4/LTB5 ratio. Overall, EΩ3FA supplementation appears effective in reducing airway inflammation in cystic fibrosis.

Similar results were seen in a study of EΩ3FA for bronchial asthma, which is also characterized by inflammatory mediation by leukotrienes [176]. Asthmatic subjects were randomized for 4 weeks to receive either 10-20 g/day of perilla seed oil (rich in ALA) or corn oil (high in Ω6) [34]. After treatment, LTB4 and LTC4 production was significantly different between groups, both of which increased in the corn oil group and decreased in the perilla group. Along with the changes in immune parameters, peak expiratory flow, forced vital capacity, forced expiratory volume, and forced expiratory flow at 25% vital capacity significantly increased in the perilla seed group compared to no change in the corn oil group. Therefore, EΩ3FA supplementation was successful in mitigating LTB4 production and airway inflammation, significantly improving several measures of asthma severity.

These results were consistent with another study by the same group of scientists using 10-20 g/day perilla seed oil in patients with asthma associated with lipometabolism [35]. However, in this study, subjects were split into two groups, depending on whether they achieved LTC4 suppression with supplementation (Group A had suppression and Group B did not). After 2 and 4 weeks of supplementation, LTC4 production significantly decreased in Group A, while it significantly increased in Group B. The peak expiratory flow value increased significantly in both groups after 2 and 4 weeks but was significantly lower in Group A during the study period. Forced vital capacity, forced expiratory volume, and forced expiratory flow at 25% vital capacity values were also significantly lower in Group A than in Group B before supplementation, indicating that they had more severe asthma at baseline. However, after treatment, these measures increased significantly after treatment in Group A and not Group B. In addition, Group A had significantly higher serum lipids at baseline, but saw significant decreases in total cholesterol, LDL-C, and phospholipids after supplementation. Therefore, supplementation with perilla seed oil was more effective in improving measures of asthma severity in those whose generation of leukotrienes was modified by EΩ3FA supplementation. Furthermore, it appears that supplementation with ALA can affect lipometabolism, which may also be associated with changes in leukotriene production.

In another study, subjects with CKD were randomized for 8 weeks to receive either 4 g/day EΩ3FA (a combination of EPA and DHA), CoQ10, or a placebo of olive oil [36] to determine the effects on LTB4 and LTB5; other chemotactic agents produced by neutrophils such as myeloperoxidase; specialized pro-resolving mediators, such as 18-hydroxyeicosapentaenoic acid (HEPE) and 14-hydroxydocosahexaenoic acid (HDHA); and metabolites of EPA and DHA, such as resolvin (Rv)E2, RvE3, and RvD5. LTB4 production did not change significantly in any group; however, LTB5 production significantly increased in both the EΩ3FA and CoQ10 groups. Plasma myeloperoxidase significantly decreased and 18-HEPE, 14-HDHA, RvE1, RvE2, RvE3, and RvD5 significantly increased in the EΩ3FA group but not the CoQ10 group. Furthermore, post-intervention LTB5 was positively correlated with platelet EPA content, 18-HEPE, 14-HDHA, RvE2, RvE3, and RvD5. Therefore, EPA and DHA supplementation appeared to increase the content of metabolites of EΩ3FA, while decreasing inflammation and increasing concentrations of specialized pro-resolving mediators, which was all associated with a decrease in the LTB4/LTB5 ratio in patients with low-grade inflammation from CKD.

In another study, hypercholesterolemic subjects were given three different diets for 6 weeks to compare PBMC cytokine responses: One rich in ALA, one rich in LA, and the standard American diet [37]. A diet rich in ALA has been shown to reduce myocardial infarction recurrence [177], protect against ischemic heart disease in women [178], and reduce coronary arterial disease (CAD) prevalence in both genders [179]. Overall, ALA appears to decrease CVD risk by reducing lipids, lipoproteins, inflammatory markers, and adhesion molecules [180]. The results of the current study add to those previous findings. Under LPS stimulation, PBMC production of IL-6, IL-1β, and TNF-α was significantly lower after consumption of the ALA diet compared to the standard American diet, and IL-1β and TNF-α production was even lower when compared to the LA diet. Furthermore, serum ALA change was significantly inversely correlated with the change in PBMC TNF-α concentration for those consuming the ALA diet. The increase in serum ALA was also correlated with an increase in PBMC ALA and EPA, both of which were then inversely correlated with PBMC production of TNF-α. Therefore, incorporation of ALA and EPA into PBMC may inhibit inflammatory cytokine release, especially of TNF-α.

3.1.2.2. Vitamin B12

Vitamin B12 is necessary for cell replication, including production of T lymphocytes. Therefore, sufficient Vitamin B12 is important in maintaining lymphocyte counts within their normal ranges, for example, a normal CD4/CD8 ratio [181]. However, Vitamin B12 deficiency is common and can cause both minor and serious clinical complications. For example, pernicious anemia is an autoimmune condition that destroys gastric parietal cells, resulting in a lack of binding between intrinsic factor and B12. Pernicious anemia is also associated with other autoimmune disorders, such as thyroid disease, diabetes, and vitiligo. Therefore, Vitamin B12 supplementation is indicated for pernicious anemia [38]. Pernicious anemia patients were given 1000 mg/day of Vitamin B12 (cyanocobalamin) intramuscularly until serum Vitamin B12 levels were within the normal range (200-900 pg/mL) [182]. At baseline, all subjects had a depressed level of Vitamin B12 (average of 85 pg/mL). After treatment, the leukocyte count significantly increased, and the lymphocyte count significantly decreased. Both absolute and relative CD4+ T cell counts increased insignificantly. However, absolute and relative CD8+ T cell counts increased significantly, leading to a nearly significant decrease in CD4/CD8 ratio, which was elevated at baseline. Absolute CD3+ (significant) and CD7+ (non-significant) T cell counts increased after treatment. The absolute and relative CD19+ B cell counts significantly increased, and the relative CD20+ cell count significantly decreased. The absolute and relative CD10+ cell counts non-significantly decreased, and the absolute CD20+ cell count non-significantly decreased. Absolute and relative CD16+ and CD56+ cell counts non-significantly increased from their depressed levels at baseline, indicating impaired NKCA. Complement component 3 (significant) and complement component 4 (non-significant) increased. IgG, IgA, and IgM all significantly increased. Thus, cyanocobalamin treatment successfully reversed the CD8+ T cell count decrease and abnormalities in B lymphocyte and NK subsets, augmenting cellular immunity. It also restored function of the complement system and boosted humoral immunity by restoring immunoglobulin concentrations. Overall, these results support the use of B12 supplementation in treating immunodeficiencies in pernicious anemia.

3.1.2.3. Vitamin D3 (cholecalciferol)

Low serum Vitamin D (25-hydroxyvitamin D (25(OH)D)) level is associated with several autoimmune disorders, such as MS [183], lupus, [184], Addison’s disease [185], and Crohn’s disease [186]. In murine models of MS, Vitamin D supplementation successfully ameliorated experimental autoimmune encephalitis [187]. IL-17 has been found to play a large role in the development of experimental autoimmune encephalitis, and a predominance of CD4+IL-17+ T (Th17) cells [188] and effector memory CD4+ T (TEM CD4+) cells [189] has been found in MS lesions. However, the effects of Vitamin D on these parameters have not been confirmed in humans.

In one study, subjects with MS were randomized for 6 months to receive either 10,400 IU/day (high dose) or 800 IU/day (low dose) of cholecalciferol to investigate the effects of Vitamin D at high and low dosages [39]. After treatment, serum Vitamin D level increased more significantly in the high-dose group compared to the low-dose group. The proportion of T-helper (Th)17 cells and TEM CD4+ cells significantly decreased from baseline in the high dose group. While only Th17 cells were significantly different between groups, the reductions in Th17 cells and TEM CD4+ cells were strongly correlated. Therefore, high-dose Vitamin D supplementation successfully decreased immune parameters that are pivotal to the development of experimental autoimmune encephalitis and sclerotic lesions.

In lupus, Vitamin D supplementation has been found to increase Treg expansion, which in turn suppresses effector T cell proliferation [190] and decreases Th1 and Th17 cells [191]. It has also been found to inhibit activation and maturation of DCs [184] and B cells [192] and immunoglobulin production [191]. Vitamin D deficient (<30 ng/mL) lupus patients with low disease activity (SLEDAI no more than 8) were given 100,000 IU/week of cholecalciferol for 4 weeks and then 100,000 IU/month of cholecalciferol for 6 months with follow-up at 2 and 6 months [40]. After treatment, serum Vitamin D increased significantly. The frequency and absolute number of CD19+ B cells significantly decreased at 2 months follow-up, the absolute number of naive CD4+ T cells significantly increased at 2 and 6 months follow-up, and the frequency of TEM CD8+ cells significantly decreased at 2 and 6 months follow-up. The frequency and absolute number of IgD+CD27+ class-switched memory B cells significantly decreased, IgD+CD27+ memory B cells significantly decreased at 2 and 6 months follow-up, and the frequency of IgD+CD27+ marginal zone-like B cells significantly increased at 6 months follow-up. The percentage and absolute number of both resting and activated memory Tregs significantly increased at 2 and 6 months follow-up. This increase was associated with greater expression of molecules associated with Treg suppression, for example, cytotoxic T-lymphocyte-associated protein (CTLA)-4, glucocorticoid-induced TNF receptor, and latency-associated peptide. The Th1 cells significantly decreased at 2 and 6 months follow-up, and interferon (IFN)-producing CD8+ T cells and Th17 cells significantly decreased from baseline to 2 and 6 months follow-up. Therefore, Vitamin D supplementation was successful in restoring B and T cell homeostasis in lupus by increasing Tregs and decreasing Th1, Th17, and memory B cells.

In Addison’s disease, autoimmune destruction of the adrenal cortex is mediated by Th cells, CD8+ T cells, and macrophages [193]. Addison’s disease patients often exhibit an altered distribution of CD4+ T cell subtypes, with an increased percentage of late-activated T cells expressing HLA-DR (a Class II major histocompatibility complex [MHC] isotype), which is required for exogenous antigen presentation [194]. Therefore, this perturbation in T cell homeostasis plays a large role in autoimmunity [195]. The Vitamin D system is intertwined in this pathogenesis, as its receptors and activating enzymes are expressed in various immune cells that influence the function (proliferation, differentiation, and cytokine release) of adaptive and antigen-presenting immune cells [196]. However, chronic treatment with glucocorticoids to manage Addison’s disease reduces the intestinal absorption of Vitamin D, making high-dose supplementation a promising therapeutic approach.

Thus, Addison’s disease patients were randomized for 3 months to receive either 4,000 IU/day of cholecalciferol or placebo [41]. Most patients were Vitamin D deficient at baseline, but serum Vitamin D and its metabolite 1,25-dihydroxyvitamin D3 (1,25-D3) significantly increased at the end of the intervention, and no patient remained deficient. The percentages of peripheral blood late-activated Th cells and late-activated CD8+ T cells significantly decreased with a significant reduction of HLA-DR density (especially on CD8+ T cells) in the treatment group compared to placebo. The percentage of Th cells correlated negatively with 1,25-D3 concentration. The percentage of monocytes within total leukocytes significantly increased. Therefore, high-dose Vitamin D supplementation in Addison’s patients was successful in restoring Vitamin D status, resulting in the regulation of activated T cells and monocytes, with a decrease in expression of the MHC isotype that facilitates the autoimmune reaction on late-activated T cells.

In Crohn’s disease, the autoimmune response against commensal bacteria occurs in the gut and subsequent inflammation occurs with T lymphocyte infiltration [197] and abnormal DC maturity and overproduction of cytokines [198]. However, exposure to 25-D3 (the less active precursor to 1,25-D3) reduces the expression of maturation markers (e.g., CD80+) and production of cytokines (e.g., IL-12) in DCs, reducing their ability to induce responses from T cells [199].

Therefore, Crohn’s patients in remission were randomized for 26 weeks to receive 1,200 IU/day of cholecalciferol or placebo [42]. Serum Vitamin D significantly increased in the treatment group compared to baseline and placebo. CD80 expression on LPS-stimulated monocyte-derived dendritic cells (mo-DC) significantly decreased, mo-DC production of IL-10, IL-6, and IL-1β significantly decreased, and mo-DC production of IL-8 decreased non-significantly compared to baseline, whereas these parameters did not change in the placebo. Stimulation of mo-DC with both LPS and Vitamin D3 resulted in an upregulation of CD14, IL-10, and IL-8, and even more so with Vitamin D3. However, this effect was attenuated significantly in the treatment group, but not in the placebo group. Therefore, treatment with Vitamin D in Crohn’s disease patients successfully reduced expression of maturation markers and production of inflammatory cytokines in stimulated mo-DC, shifting mo-DCs toward an anti-inflammatory state. However, the inhibition of cytokine production was not specific, but rather an overall general reduction. Nonetheless, Vitamin D supplementation demonstrates some promise in decreasing disease severity in Crohn’s disease and other autoimmune conditions.

Vitamin D supplementation has also been used as a treatment for cancer. Studies in head and neck squamous cell carcinoma (HNSCC) patients reveal that infiltration of CD4+ and CD8+ T cells correlates with prognosis [200]. However, immune dysfunction in these patients is facilitated by elevated levels of, and defects in, suppressive CD34+ cells, preventing them from maturing into DCs and stimulating naive T cells [201]. Furthermore, studies demonstrate that HNSCC patients have a low level of mature DCs and the presence of DCs that express markers of immaturity [44]. However, Vitamin D3 (1,25-D3) has been found to induce CD34+ cells’ maturation into DCs, decreasing the intratumoral level of CD34+ cells (25).

Thus, HNSCC patients were treated orally for three cycles (3 weeks total) with 4 mg/day of Vitamin D3 (1,25-D3) for 3 days followed by 4 days without treatment or no treatment before undergoing surgery for their cancer [43]. After 3 weeks of treatment, intratumoral CD4+ and CD8+ T cell counts had 3-fold and 4.5-fold increases, respectively. In addition, the infiltrating cells expressing CD69 (an activation marker) increased by approximately 10-fold. Along with these immunological changes, those who received Vitamin D3 (1,25-D3) had a significantly longer time to recurrence compared to control. Therefore, pre-surgical treatment with Vitamin D3 (1,25-D3) increased intratumoral T cell infiltration (possibly through decreased CD34+ count) and early activation marker expressing cells with a concomitant reduction in HNSCC recurrence rate.

HNSCC patients were studied in a similar trial using the same treatment cycles and length but with 4 mg/day of enteric Vitamin D3 (1,25-D3) or no treatment [44]. After 3 weeks, the intratumoral CD34+ cells and DCs expressing immature markers significantly decreased, and the intratumoral mature DCs significantly increased in the treatment group compared to control. These results corroborate those of the previous study and further support the use of Vitamin D3 as a therapy for HNSCC.

In another study, HNSCC patients were randomized for 6 weeks to receive either 20, 40, or 60 μg/day of Vitamin D3 (25-D3) [45]. Treatment resulted in a significant decrease in the CD34+ cell level in those with a high baseline CD34+ cell count (>1%), but not those with low CD34+ cell counts in the 40 and 60 μg groups. This effect was the most consistent and persistent in the 60 μg group and was evident by the end of week 1. HLA-DR expression and plasma IL-12 in the high CD34+ cell patients increased in a dose-dependent manner, and the change in HLA-DR occurred toward the later stages of treatment. T cells from low CD34+ patients showed greater potential for blastogenesis than those from high CD34+ patients, but the T cell blastogenesis in high CD34+ patients increased in a dose-dependent manner, with only the 60 μg group achieving significance. Plasma IFN-γ significantly increased for all doses, with the most prominent increase occurring in high CD34+ cell patients at week 6 and no significant changes in low CD34+ cell patients. Therefore, treatment with lower doses (20 and 40 μg) resulted in early temporary decreases in CD34+ cell count, but a higher dose (60 μg) led to a more enduring decrease. Treatment at higher doses (40 and 60 μg) also caused increases in MHC II expression, plasma IL-12 and IFN-γ, and T cell blastogenesis. These effects were mostly more pronounced in those with higher CD34+ cell counts, illustrating promise for higher dose Vitamin D3 (25-D3) supplementation in these patients.

In addition to HNSCC, Vitamin D supplementation has also been investigated in colorectal cancer (CRC), the severity of which is inversely associated with serum Vitamin D levels [202]. Inflammatory markers such as CRP, TNF-α, and IL-6 are associated with higher tumor grade and risk of mortality in CRC patients and are elevated in the blood of CRC patients compared to healthy controls [203]. However, Vitamin D receptor activation is involved in the regulation of inflammatory cytokine production, cell cycle regulation, and apoptosis [204], giving it potential in treating CRC.

CRC patients were randomized for 6 months to receive either 800 IU/day of Vitamin D3 or placebo [46]. Vitamin D3 treatment resulted in a significant 60% increase in serum Vitamin D and significant decreases in CRP (32%), TNF-α (13%), IL-6 (32%), IL-1β (50%), IL-8 (15%), and an overall inflammation z-score (77%) compared to placebo. Changes in CRP and the overall inflammation score were more pronounced in men than women and those who did not use nonsteroidal anti-inflammatory drug (NSAIDs), compared to NSAID users. Therefore, Vitamin D3 may decrease colonic inflammation, which reduces the risk of CRC neoplasms. Overall, Vitamin D3 supplementation appears to be an effective therapy for multiple cancers, warranting ongoing investigation.

Vitamin D has also been investigated in counteracting low-grade inflammation in chronic inflammatory conditions, for example, T2DM and arthritis. In T2DM, adipokines have been shown to influence both insulin activity and inflammation [205]. For example, adiponectin has been found to favor glucose uptake in muscle and inhibit gluconeogenesis in the liver, yielding improvements in insulin sensitivity [206], while retinol-binding protein-4 has been found to impair insulin sensitivity and is often elevated in those with T2DM [207]. Cytokines have also been found to strongly predict the development of T2DM [208]. For example, TNF-α and IL-1 are believed to reduce adiponectin and lead to abnormal glucose tolerance [209]. Therefore, the previously established ability of Vitamin D to mitigate pro-inflammatory cytokine production gives it potential in T2DM.

Subjects with T2DM were randomized for 12 weeks to receive 2 bottles/day of a yogurt drink, either fortified with 500 IU of cholecalciferol or with no detectable cholecalciferol [47]. The Vitamin D group showed an improvement in Vitamin D status, validating the bioavailability of Vitamin D in the yogurt drink. Those who received Vitamin D also saw significant decreases in CRP, IL-1β, IL-6, retinol-binding protein-4, and fibrinogen and significant increases in serum IgM and adiponectin. Since natural IgM antibodies have been found to protect against atherosclerosis [210], this finding, along with the decrease in fibrinogen, also has implications for additional metabolic conditions characterized by vascular dysfunction. Therefore, modulations in adipokines and markers related to vascular dysfunction and improved inflammatory status highlight the ability of Vitamin D supplementation to improve insulin sensitivity and decrease the risk of atherosclerosis in subjects with T2DM.

Treatment with Vitamin D has also been successful in experimental arthritis models, showing suppression of arthritis incidence and activity [211]. However, these results have not been demonstrated in adults with psoriatic arthritis. Inflammation in psoriatic arthritis is primarily mediated by IFN-γ, as it increases MHC-II expression on ICAM-1 on the surfaces of synovial membranes, facilitates inflammatory cytokine production, and causes Th1 dominance while decreasing T suppressor activity [212].

Vitamin D has also proven beneficial in ameliorating inflammation and improving outcomes in procedures such as dialysis. Patients with CKD often have low Vitamin D status or disorders of Vitamin D metabolism [213] due to decreased activity of renal 1-a hydroxylase enzyme (CYP27B1), which is responsible for the conversion of 25-D3 to 1,25-D3 [214]. Furthermore, chronic inflammation is prevalent in CKD, which is associated with poor outcomes and cardiovascular events, especially in combination with low serum Vitamin D [215]. Therefore, high-dose Vitamin D supplementation should be considered for dialysis patients.

Peritoneal dialysis and hemodialysis patients were randomized for 12 weeks to receive either 50,000 IU of cholecalciferol twice/week or placebo [48]. Serum Vitamin D significantly increased (surpassing 30 ng/mL in all subjects) in the treatment group with no change in the placebo group. CRP and IL-6 significantly decreased, and monocyte Vitamin D receptor and CYP27B1 expression significantly increased in the treatment group compared to the control group. Therefore, high-dose Vitamin D supplementation was successful in restoring Vitamin D status, reducing inflammation, and improving Vitamin D metabolism in dialysis patients, potentially decreasing risk of complications.

3.1.2.4. Zinc

Zinc has been found to decrease oxidative stress by inhibiting nicotinamide adenine nucleotide phosphate (NADPH) oxidase, thus reducing ROS production. Furthermore, the antioxidant enzyme SOD contains zinc [216]. However, zinc deficiency is prevalent and can cause immune dysfunction and cognitive impairment. Deficiency of zinc also occurs in multiple diseases, such as sickle cell disease and chronic liver and renal diseases [217]. Sickle cell disease is characterized by elevated serum TNF-α and Th2 dominance [218], possibly exacerbated by zinc deficiency. Furthermore, common elevations in soluble adhesion molecule concentrations in sickle cell disease [219] can play a large role in vaso-occlusive events in sickle cell disease, which cause pain and organ dysfunction [220].

Thus, sickle cell disease patients were randomized for 3 months to receive either 25 mg zinc acetate 3 times/day or placebo [52]. The incidence of infection decreased in the zinc group with significant differences between treatment groups for total number of infections and upper respiratory tract infections. Treatment also resulted in significant increases in red blood cell, hemoglobin, and hematocrit levels compared to the placebo group. Compared to a group of healthy controls, sickle cell disease patients had lower plasma zinc, increases in markers of oxidative stress, elevated VCAM-1, increased production of TNF-α and IL-1β, and decreased IFN-γ production at baseline. After treatment, plasma zinc level and IFN-γ production significantly increased, plasma nitrogen oxide significantly decreased, and plasma MDA, hydroxyalkenals (HAE), 8-hydroxy-2’-deoxyguanosine (8-OHdG), sVCAM-1, sICAM1, TNF-α, and IL-1β decreased compared to both baseline and placebo. In addition, changes in plasma zinc were inversely correlated with TNF-α production, MDA, HAE, and 8-OHdG. Plasma zinc was also weakly positively correlated with the change in hematocrit. In addition, isolated LPS-stimulated PBMC production of cytokines was measured, with significant decreases in LPS-induced TNF-α and IL-1β mRNAs in the zinc group and not the placebo group. As a model of oxidative stress, TNF-α induced activation of NF-κB in isolated PBMCs was also measured, with a significant decrease in NF-κB DNA binding for the zinc group and not the placebo group. In addition, PHA induction of IL-2 and IL-2Ra mRNA significantly increased in the zinc group only. Therefore, zinc supplementation shows promise in zinc-deficient sickle cell disease patients, as it successfully increased erythropoiesis, decreased infection incidence, improved Th1 function, decreased soluble adhesion molecule concentrations, and decreased oxidative stress, while increasing antioxidant capacity.

Patients undergoing hemodialysis often exhibit a low level of plasma zinc [221], which can increase their propensity for inflammation and immunodeficiency, subsequently increasing mortality risk [222]. These patients also have an elevated level of plasma copper, resulting in an increased copper/zinc ratio, which is associated with the same risks and often suggested as a cause of adverse outcomes [223]. Chronic inflammation can also disrupt T cell function by decreasing the CD4/CD8 ratio and CD19+ cell counts, thereby increasing mortality risk [224]. Therefore, zinc supplementation demonstrates promise in hemodialysis patients.

In one study, hemodialysis patients were randomized for 8 weeks to receive 11 mg/day of elemental zinc or no treatment [53]. At baseline, patients had significantly lower plasma zinc, SOD activity, percentages of CD3+ and CD4+ T cells, CD19+, CD4/CD8 ratio, and higher plasma copper, copper/zinc ratio, MDA, CRP, TNF-α, and IL-1β compared to healthy subjects. After treatment, the zinc group trended toward an increase in plasma zinc, showed decreases in copper and the copper/zinc ratio, had significant decreases in MDA, CRP, TNF-α, and IL-1β, and significant increases in SOD activity, percentages of CD4+ T cells and CD19+, and non-significant increases in CD4/CD8 ratio compared to baseline and control. In addition, the copper/zinc ratio was positively correlated with MDA concentration, CRP, TNF-α, and IL-1β. Therefore, zinc supplementation in hemodialysis patients helped to restore plasma zinc concentration and normalize the copper/zinc ratio, resulting in a decrease in oxidative stress, an increase in antioxidant activity, and normalization of T cell function, likely decreasing morbidity and mortality risk.

HIV is a condition in which zinc deficiency affects immune function by reducing T cell production and causing lymphopenia, resulting in an increase in infection incidence [225]. When CD4+ T cell count drops below 200 cells/mm^3^, PLWH are susceptible to infection, even with ART, and have an increased risk of mortality [226]. In one study, PLWH were randomized for 18 months to receive zinc (12 mg/day for females and 15 mg/day for males) or a placebo [54]. After treatment, those who received zinc had significantly higher plasma zinc compared to control. The change in viral load was not significant. However, zinc treatment still prevented immunological failure (i.e., a drop in CD4+ T cell count below 200 cells/mm^3^), meaning that a 4-fold decrease in the likelihood of immunological failure occurred compared to the placebo, when controlling for confounders. Therefore, zinc supplementation was able to provide protection against lymphopenia and subsequent infection, despite persisting viral load. Furthermore, adherence to the zinc supplementation regimen was higher than that to ART, making it an effective treatment.

Diabetes is another condition characterized by abnormal zinc homeostasis, perhaps due to impaired absorption or elevated urinary excretion [227], and low plasma zinc concentration is associated with diabetic complications [228]. A high concentration of TNF-α is associated with insulin resistance [229], but transmembrane TNF-α has been found to be involved in defense against infection and cancer [230]. Zinc supplementation has been found to stimulate PBMC TNF-α production [231] and LPS-stimulated monocyte cytokine production [232], and diabetic patients have experienced decreases in monocyte derived (transmembrane) TNF-α in response to stress [233]. Therefore, zinc has potential in augmenting immune function in T2DM.

In one study, T2DM patients with MetSyn were randomized for 8 weeks to receive either 30 mg/day of elemental zinc or placebo [55]. With treatment, plasma TNF-α did not change in either group, but transmembrane TNF-α expression on monocytes significantly increased compared to both baseline and placebo. Therefore, an increase in monocyte transmembrane TNF-α, with no concomitant increase in circulating TNF-α, implies a positive immune impact in T2DM patients, possibly decreasing cancer risk.

### 3.2. Phytonutrients

#### 3.2.1. Aloe vera polysaccharides

Aloe polymannose is an oligosaccharide found to promote CD14+ monocyte proliferation in peripheral blood [234]. CD14+ cells have stem cell-like abilities, differentiating into neurons and other cells [235]. Acemannan is a well-studied key aloe polysaccharide shown to significantly increase white blood cells, macrophages, cytokine release, cytotoxicity of T cells, and dendritic cells, thus showing its multifaceted immunomodulatory activity, which would be necessary for many different health challenges [236,237].

Thus, aloe polymannose shows potential as a therapy in cases of cognitive and immune dysfunction, such as Alzheimer’s disease (AD), which is characterized by chronic inflammation and neurodegeneration, marked by increased production of inflammatory cytokines, such as TNF-α [238]. Neuronal loss in AD is partially attributed to abnormal endothelial activation with increases in the production of endothelial markers, such as VEGF [239]. Moreover, AD is characterized by T cell abnormalities such as immunodeficiency-related [240] and age-related [241] decreases in the CD3+CD4+/CD3+CD8+ ratio.

In one study, the effects of aloe polymannose were investigated in subjects diagnosed with AD [56]. Subjects were given 4 teaspoons/day of an aloe polymannose multinutrient complex for 12 months and saw significant increases in CD14+ cells, implying a potential for neuronal regeneration. The study also noted significant decreases in TNF-α and VEGF. Finally, T cell and B cell activity significantly decreased, and the CD3+CD4+/CD3+CD8+ ratio significantly increased. Notably, these changes were correlated with increases in the Alzheimer’s Disease Assessment Scale-Cognitive Subscale cognition and concentration scores, showing that the aloe polymannose multinutrient complex successfully improved cognitive function through decreases in inflammation and endothelial dysfunction and modulation of lymphocyte activity.

In a similar study, 15 subjects with relapsing-remitting MS were given a broad-spectrum of aloe polysaccharide supplements for 1 year [57]. Cytokines, growth factors, T and B cell subsets, and the occurrence of infections were assessed at baseline and 12 months. At the end of the study, participants had significantly fewer total infections. At 12 months, IL-2, TNF-α, epidermal growth factor (EGF), and CD95+CD34+ significantly increased, while IL-1β significantly decreased. Thus, decreased infections and an overall net improved immunomodulatory profile suggested a benefit to the aloe polysaccharide-based dietary supplement regimen.

#### 3.2.2. Beta-glucans

Beta-glucans are polysaccharides found in oat and barley grains, baker’s yeast, and mushrooms. Receptors for beta-glucans are found in multiple cell types such as macrophages, NK cells, neutrophils, T cells, and endothelial cells [242], and they therefore likely play a role in immunomodulation. Beta-glucans have been used in the treatment of respiratory tract infections (RTI) in many studies.

In one study, the effects of beta-glucans were investigated in children with recurring RTI (RRTI), characterized as more than five RTIs within the 12 months before the trial [58]. Subjects were randomized to receive either 1 mL/5 kg/day for 6 months of either: treatment syrup (10 mg beta-glucans derived from mushrooms with 10 mg Vitamin C/mL) or placebo syrup (only 10 mg Vitamin C/mL) and were assessed at baseline, after 6 months of treatment, and 6 months post-treatment. Overall, the treatment group demonstrated total clinical efficacy with a significant decrease in RTI incidence. The number of CD19+ B lymphocytes decreased in both groups after 12 months. In the treatment group, IgG concentration increased during treatment and remained elevated, while no change occurred in the placebo group. The same pattern occurred with IgM concentration. Further, both groups saw an increase in IgA concentration over 12 months, but the difference between baseline and 12 months was higher in the beta-glucans treatment group. Increases in all three Ig isotypes demonstrate beta-glucans’ ability to bolster the humoral innate immune response, with changes remaining past the supplementation period. The number of CD3+, CD4+, and CD8+ T-lymphocytes decreased throughout the study in both groups, but the beta-glucans’ group had a slower decline in the number of CD8+ T cytotoxic lymphocytes, revealing maintenance of the necessary adaptive immune response. In addition, the NK cell count increased in the beta-glucans group throughout the administration of the treatment syrup. However, the NK cell count increase returned to baseline after 6 months without administering treatment syrup. Overall, treatment with beta-glucans showed improvements in innate and adaptive immunity, while improving respiratory morbidity with no adverse effects.

Beta-glucans have also been used as a therapy in breast cancer (BCa) in multiple studies. Beta-glucans have been found to stimulate macrophages and neutrophils, promoting cell-mediated immunity [243]. CD95, associated with macrophage activation, is a cell surface molecule that regulates immune responses through apoptotic signaling [244]. CD45RA is another monocyte activation marker and is correlated with illness severity [245]. Therefore, beta-glucans’ ability to activate these phagocytic cells, potentially upregulating CD95 and CD45RA, gives them potential as an adjunct therapy to compliment antibody-based cancer therapies.

In a study of women with newly diagnosed or relapsed metastatic BCa, subjects were given two 10 mg capsules/day for 14 days of baker’s yeast-derived beta-glucans [59]. At baseline, the BCa subjects had a significantly lower lymphocyte count and CD95 expression on CD14+ cells compared to the healthy controls. After 14 days of beta-glucans therapy, both mean and relative monocyte count increased. Expression of both CD45RA on CD14+ cells and CD95 also increased significantly. Overall, the beta-glucans treatment appeared to increase the proliferation and activation of peripheral blood monocytes with no adverse effects.

In addition to lymphopenia, cancer patients often present with leukopenia, that is, a decrease in white blood cell (WBC) count. However, it has been previously reported that beta-glucans can stimulate hematopoiesis [246], giving the promise to alleviate this immunosuppression source. Cancer is also associated with the perturbation of the balance between Th1 and Th2 cytokine production or the Th1/Th2 ratio. A balanced Th1/Th2 ratio is crucial in anti-tumor immunity, as Th1 shows robust anti-tumor activity [247], and Th1 and Th2 cells can inhibit each other’s functions through cytokine production [248].

In another study, women with BCa were randomized for 21 days to receive either two 10 mg capsules/day of baker’s yeast-derived beta-glucans or placebo in between chemotherapy courses to investigate the effects on WBC count and the proportion of production of IL-4, a Th2 cytokine, and IL-12, a Th1 cytokine [60]. In both groups, WBC count decreased, but with a slightly milder decline in the beta-glucans group. Serum IL-4 level significantly decreased in the beta-glucans group compared to placebo, and IL-12 increased in the beta-glucans group, while it decreased in the control group compared to baseline. Therefore, it appears that beta-glucans supplementation not only ameliorated leukopenia but it also shifted the immune response toward Th1 cytokines and away from Th2 cytokines, allowing for a greater anti-tumor immune response.

Beta-glucans have also been shown to modulate the Th1/Th2 balance as an immunoprotective effect in other conditions. In one study, beta-glucans were evaluated for their effects on allergic rhinitis (AR), an IgE-mediated disease [61]. IgE-mediated diseases result from Th2 cell dominance, leading to Th2 cytokine overproduction [249]. Olive pollen-sensitized AR patients were randomized for 12 weeks to receive either 10 mg beta-glucans twice/day or placebo, followed by a nasal provocation test using olive pollen and a sampling of nasal lavage fluid. After treatment, the beta-glucans group saw significant decreases in nasal lavage fluid containing IL-4 and IL-5 (Th2 cytokines) levels, while IL-12 (Th1 cytokine) significantly increased, and INF-g did not change. No biomarkers changed in the placebo arm. Therefore, beta-glucans were successful in decreasing Th2 cytokine levels while increasing Th1 cytokine levels. In addition, the percentage of eosinophils, a cell responsible for mediating allergic responses by binding IgE, decreased significantly in the beta-glucans treatment group [250], with no change in the placebo group. Therefore, beta-glucans’ ability to shift the immune system toward a Th1-mediated response is well-established and useful in targeting multiple different pathologies.

The ability of beta-glucans to stimulate NKCA [251] also holds potential in critically ill patients, such as those in the intensive care unit (ICU), who often have hyporeactive NK cells [252]. Beta-glucans have also been found to increase activation of macrophages [251], which then release IL-12. This increase in IL-12 allows for greater phagocytosis and general cell-mediated immunity [253].

In a study of ICU patients with either pulmonary disease or trauma, subjects were randomized to one of three enteral nutrition arms: Standard enteral nutrition, immune-modulating nutrients with 50 mg beta-glucans/200 kcal (derived from mushrooms), or immune-modulating nutrients without beta-glucans [62]. After 7 days, the beta-glucans group showed significant increases in NKCA compared to the control group. PBMC IL-12 release and serum IL-12 concentration slightly increased in the beta-glucans group, whereas PBMC IL-12 release significantly decreased in the control group. Furthermore, the changes in PBMC IL-12 and NKCA were positively correlated, suggesting a strong immunoprotective effect of beta-glucans. Serum CRP decreased in both the beta-glucans and immune-modulating nutrients groups compared to the control group but decreased more in the immune-modulating nutrients group. Changes in CRP and PBMC IL-12 were negatively correlated, showing that the increases in NKCA and macrophage activity were not accompanied by more inflammation. Overall, these results point to beta-glucans as a potential therapy for critical illnesses by increasing cell-mediated immunity without causing additional low-grade inflammation.

#### 3.2.3. Bilberry

Bilberries are rich in polyphenols [254], which have antioxidant properties and have been found to be beneficial in preventing chronic inflammation and disease, such as CVD [255]. Systemic inflammation in CVD is associated with increased production of cytokines and other inflammatory markers such as CRP and is associated with the activation of NF-κB, a known mediator of multiple inflammatory responses and pathways [256]. Subjects at risk for CVD were randomly assigned to drink either 330 mL/day of bilberry juice (diluted to 1 L using tap water) or 1 L/day of water, in addition to typical fluid consumption [63]. The bilberry group saw significant decreases in plasma CRP, IL-6, IL-15, and monokine induced by IFN-γ compared to the water group. TNF-α increased in the bilberry group; however, the TNF-α:IL-10 ratio did not change significantly, indicating no substantial increase in inflammation. Subjects with features of MetSyn were randomly assigned to consume either a bilberry-rich diet (equivalent to 400 fresh bilberries) or to eat *ad libitum* [64]. Similar to CVD, MetSyn is characterized by the overproduction of several inflammatory markers [257]. Inflammation is exacerbated by the increased expression of genes, such as C-C motif receptor 2, which attracts monocytes to sites of inflammation and facilitates the differentiation of monocytes into macrophages [258]. After 8 weeks, the bilberry group saw a significant decrease in IL-6, IL-12, CRP, and LPS levels compared to the control. Therefore, these subjects experienced an overall decrease in low-grade inflammation, suggesting the potential to improve chronic disease risk in individuals with features of MetSyn.

In addition, these changes correlated with significant decreases in mRNA expression of C-C motif receptor 2 and monocyte to macrophage differentiation. This would indicate an increase in humoral immune responses and a concurrent decrease in the innate immune responses often seen in low-grade inflammation and MetSyn. Overall, bilberry supplementation appears to decrease low-grade inflammation by lowering circulating levels of inflammatory biomarkers, providing great therapeutic potential for conditions such as CVD and MetSyn by mitigating the risk of atherosclerosis and endothelial dysfunction.

#### 3.2.4. Black seed oil

Black seed oil contains thymoquinone, which exhibits antioxidant effects through inhibition of pro-inflammatory pathways [259]. Rheumatoid arthritis (RA) is an autoimmune disease that causes swollen and painful joints. It is characterized by inflammation and concomitant increases in neutrophil recruitment, leading to elevated WBC [260]. In addition, RA causes dysregulation of glutathione, which generally protects against ROS [261]. Thus, black seed oil has the potential to alleviate the inflammation seen in RA.

RA patients were given 500 mg black seed oil twice/day for 1 month and saw a significant decrease in WBC count to within a normal level [65]. In addition, these patients saw an increase in blood glutathione level. Along with these changes, patients reported decreases in morning stiffness and the number of swollen joints. Thus, black seed oil shows potential as an adjunct therapy for RA.

Black seed oil has also been investigated in naturopathic allergy treatment, as it has been shown to inhibit histamine release from mast cells [262]. Histamine release is often mediated by IgE, which not only controls inflammatory mediators but has also been found to play a role in tissue modeling with chronic exposure to allergens [263]. One study investigated the effects of black seed oil on patients with allergic rhinitis [66]. Patients took three or four 500 mg black seed oil capsules (depending on body weight) twice/day for 28 days. No significant changes occurred in lymphocytes or lymphocyte subpopulations (T lymphocytes, Th cells, T-suppressor cells, B lymphocytes, or NK cells). However, the mean score of subjective symptom severity decreased continuously and significantly throughout the treatment. These results imply that black seed oil has the potential for alleviating the severity of allergic symptoms.

Another study investigated black seed oil supplementation during pregnancy in women with atopic dermatitis, measuring its effects on the mother’s atopic status and the development of the condition in the infant [67]. Mothers were randomized to receive either black seed oil or placebo during the 8^th^-16^th^ week of pregnancy, continuing until after 3 months of breastfeeding. Breast milk contains antibodies and lymphocytes that can protect an infant against infections [264], which is especially crucial within the 1^st^ year of infancy when the atopic immune response is developed. Oversensitization to allergens often occurs in neonates whose Th1 response (e.g., IFN-γ) is not adequately developed *in utero* to suppress the production of inflammatory Th2 cytokines (e.g., IL-4 and IL-5) [265]. Thus, the study measured breast milk Th1 and Th2 cytokine levels with and without black seed oil intervention. The level of IL-4 in breast milk was significantly lower in the black seed oil group compared to placebo, while the level of IFN-γ was significantly higher. Therefore, black seed oil supplementation enhanced the Th1 response while suppressing the Th2 response in breast milk, possibly decreasing the infant’s chance of developing atopic dermatitis.

#### 3.2.5. Curcumin (turmeric)

Curcumin, the most biologically active component of the turmeric root (*Curcuma longa*), has been demonstrated to be an anti-inflammatory agent via inhibition of lipoxygenases, COX-2, leukotrienes, prostaglandins, and cytokines, among others [266]. Curcumin has been evaluated in the treatment of autoimmune diseases, such as MS. In addition to T cell migration, autoreactivity in MS is also characterized by Treg dysfunction, which occurs with inflammation [267]. Tregs provide defense against infections and tumor cells [268], in part by secreting IL-10 and TGF-b [269]. TGF-b typically induces FoxP3 protein expression and Treg differentiation, which helps in immune tolerance, but is impaired in MS [270].

MS patients were randomized to receive either a daily dose of 80 mg of nanocurcumin or a placebo for 6 months [68]. The nanocurcumin group saw increases in Treg frequency, FoxP3 expression, and PBMC expression and secretion of TGF-b and IL-10 compared to the placebo group. Thus, curcumin improved several immune markers that are normally impaired in MS. A similar study was conducted in MS patients who were randomized to receive either a daily dose of 80 mg of nanocurcumin or a placebo for 6 months to evaluate the change in microRNA expression [69]. MicroRNAs are involved in hematopoiesis and T cell function, as they can suppress various messenger (m)RNAs. The autoreactivity in MS can be partially attributed to microRNA dysfunction, and they are expressed differently in MS patients compared to healthy individuals [271]. MicroRNAs were measured at pre-treatment (in MS patients and healthy controls) and after treatment (in MS patients only). At baseline, 13 microRNAs in CD4+ T cells (miR-16, miR-17-5p, miR-17-92, miR-27, miR-29b, miR-126, miR-128, miR-155, miR-326, miR-550, and miR-340) and miR-132 in B cells were overexpressed, implying that they are likely dysregulated and involved in the pathogenesis of MS. After 6 months of treatment, every microRNA expression significantly decreased in the previously overexpressed T cells compared to placebo. At baseline, 16 microRNAs in PBMCs were downregulated (miR-15a, miR-16-1, miR-18a, miR-20b, miR-25, miR-106b, miR-363, miR-31, miR-181c, miR-374a and miR-150 in T cells, and miR-16, miR-19b, miR-320a, miR-340, and miR-599 in B cells). The expression of nine of these microRNAs (miR-15a, miR-16, miR-19b, miR-106b, miR-320a, miR-363, miR-31, miR-150, and miR-340) significantly increased after 6 months of treatment. Thus, curcumin treatment restored most of the aberrant microRNA expression, normalizing T and B cell function, without adverse effects.

Curcumin was also investigated as a possible therapy for osteoarthritis (OA) patients, who were randomized to receive either 30 mg curcuminoids 3 times/day or 25 mg diclofenac sodium 3 times/day [70]. Diclofenac sodium is an NSAID commonly used to treat joint stiffness through inhibition of macrophage COX-2 production in synovial fluid [272]. However, it has been established that curcumin also inhibits COX-2 production, as well as prostaglandins, MCP-1, TNF-α, and IL-12, which all contribute to inflammation, and thus joint stiffness in OA [266]. The two treatments were compared for their ability to reduce COX-2 secretion by synovial fluid monocytes, ultimately finding no significant difference between them. However, since NSAID usage is often associated with impairment of the gastrointestinal, renal, and cardiovascular systems [273], curcumin is perhaps a superior OA treatment in that it alleviates COX-2-mediated inflammation equally, but without adverse effects.

Curcumin was also explored as a treatment for diabetic kidney disease in T2DM. Diabetic kidney disease is partially caused by hyperglycemic oxidative stress and inflammation. Usually, anti-oxidative capacity is mediated by the nuclear factor erythroid-derived 2-like 2 (Nrf2) system, but this capacity is blunted in inflammation caused by T2DM [274]. However, curcumin appears to be a strong activator of Nrf2, making it effective in combating diabetic kidney disease and other complications [275]. In one study, T2DM patients took 500 mg/day of curcumin for 15 days, and those with diabetic kidney disease received treatment for an extra 30 days [71]. After treatment, plasma MDA decreased significantly, and proteins associated with Nrf2 system function (i.e., NADPH quinone-1, SOD1, and SOD2) significantly increased. These biomarkers changed to a greater extent in those with diabetic kidney disease, but not significantly. Furthermore, LPS significantly decreased, indicating lowered inflammatory status. In addition, IkB kinase, a protein that inhibits NF-κB to mitigate inflammation, increased [276]. These changes were accompanied by a considerable reduction in urinary albumin excretion, which is an important measure of diabetic kidney disease severity. Therefore, it appears that curcumin can help to combat the inflammation in T2DM and diabetic kidney disease by activating the Nrf2 system and suppressing inflammatory signaling.

#### 3.2.6. Frankincense (Boswellia serrata)

Frankincense, or *B. serrata*, is commonly used in traditional Eastern medicine. The plant’s boswellic acids have immunomodulatory properties, through inhibition of inflammatory enzymes, NF-κB activity, and inflammatory cytokine signaling [277,278]. Therefore, *B. serrata* has the potential as a phytotherapy for inflammatory diseases. MS patients were treated with boswellic acids for 8 months with individual doses determined by tolerability (minimum tolerated dose was 2400 mg/day to continue in the trial) [72]. Patients were also given the option to extend treatment for up to 36 months, and 18 out of 38 patients accepted. TGF-b, IL-4, and IL-5 significantly decreased at months 1, 3 (early treatment), 8t, and 12 (late treatment). IL-17A, IL-2, and IFN-γ significantly decreased only during late treatment. In addition, the number and volume of lesions decreased on magnetic resonance imaging. In the patients who chose the extended treatment, reduced disease activity was maintained for up to 36 months. Mild to moderate gastrointestinal adverse events occurred in some subjects, especially during the first 4 weeks of treatment. Thus, boswellic acids appear to decrease inflammation and imaging outcomes successfully but are not entirely well-tolerated.

Boswellic acids were also investigated as a potential treatment to improve outcomes in ischemic stroke patients, as the overproduction of inflammatory cytokines is implicated in the pathogenesis of stroke [279]. The overproduction of cytokines and chemokines tends to accelerate inflammation by recruiting leukocytes and modulating blood-brain barrier permeability [280,281]. More specifically, TNF-α, IL, and prostaglandins have been found to exacerbate ischemic inflammation in the brain [282]. In one study, ischemic stroke patients were randomized to receive either two 400 mg capsules of boswellic acids or placebo 3 times/day for 30 days [73]. Within 7 days of treatment, plasma TNF-α and prostaglandin E2 (PGE2) significantly decreased in the boswellic acid group compared to placebo. Plasma MCP-1, IFN-γ inducible protein-10, and IL-8 also significantly decreased in the boswellic acid group compared to placebo. In addition, the National Institutes of Health Stroke Scale score, which measures neurologic deficit severity, improved. Therefore, boswellic acid supplementation may improve ischemic stroke risk and outcomes by mitigating the release of inflammatory mediators.

#### 3.2.7. Garlic

A higher intake of alliums, such as garlic, is associated with a reduced risk of CVD [283]. This risk reduction is partially due to their sulfur-containing compounds (e.g., allicin), which are hypothesized to have anti-atherosclerotic properties [284]. It has also been found that garlic can influence blood lipid profiles by increasing cholesterol efflux [285], which is inversely related to CVD risk [286].

In one study, patients with CAD who underwent angioplasty were randomized for 3 months to receive either twice-daily garlic tablets containing 1200 mg of allicin/tablet or placebo beginning 3 days after angioplasty [74]. Flow mediated dilation in the brachial artery significantly improved, indicating better endothelial function. CRP significantly decreased after treatment, and it was negatively correlated with cholesterol efflux, which did not change at the end of the study. However, study participants already had optimal cholesterol levels, which would likely explain why cholesterol efflux did not change. Nonetheless, the relationship between CRP and efflux shows some promise for garlic as a CAD therapy, with more research warranted for those with hypercholesterolemia.

Obesity is often associated with an increased risk of systemic inflammation [287] through production of TNF-α and IL-6 from adipocytes [288]. The inflammation is accompanied by reduced numbers of NK T cells [289] and gamma delta (gd) T cells [290] and thus impaired immune function. The immunomodulatory effects of garlic were investigated in obese adults, with participants randomized for 6 weeks to receive either three 0.6 g aged garlic extract capsules twice daily or placebo [75]. At baseline, NK T cells made up a higher proportion of the lymphocyte population (potentially because they migrated from adipose into circulation), while γδ T cells were suppressed. After treatment, the aged garlic extract group had a significantly lower percentage of NK T cells and a higher percentage of γδ T cells compared to placebo. The aged garlic extract group also had significantly lower IL-6 and TNF-α levels which were elevated at baseline compared to placebo. Therefore, garlic supplementation was successful in restoring immune cell counts and mitigating the systemic inflammation that occurs with obesity.

Like obesity, cancer is another disease with lowered immune cell counts. Immune dysfunction is common in cancer, making patients even more susceptible to cancer proliferation and infection. However, some research suggests that garlic may suppress carcinogenesis [291]. Thus, patients with advanced cancer of the colon, liver, or pancreas were randomized for 3 months to receive four 500 mg aged garlic extract capsules/day or placebo [76]. Salivary cortisol increased significantly in the control group but did not change in the aged garlic extract group. NKCA and count increased significantly in the aged garlic extract group but not in the control group. Furthermore, five subjects in the control group had a rapid decrease in NKCA during the study, while none did in the aged garlic extract group, and three of these five subjects died in the following 3 months. Therefore, aged garlic extract supplementation significantly increased NKCA and count and may have contributed to lower mortality risk in the treatment group.

#### 3.2.8. Ginger

Ginger has been evaluated in many clinical trials for its ability to alleviate nausea and vomiting, but it also has multiple effects on immune functioning. Ginger contains over 40 antioxidant compounds, which can be used to treat various inflammatory conditions [292]. T2DM is characterized by low-grade inflammation and increased circulation of inflammatory cytokines, contributing to insulin resistance [293]. Thus, the gingerols, shogaols, and diarylheptanoids in ginger may alleviate inflammation by inhibiting COX, decreasing the production of prostaglandins and cytokines [294]. T2DM patients were randomized for 12 weeks to receive either 1600 mg/day of ginger or placebo [77]. After treatment, CRP, TNF-α, and PGE2 significantly decreased in the ginger group compared to placebo. Indices of insulin resistance (glycated hemoglobin, insulin, and insulin resistance) and lipids (triglycerides, total cholesterol, and high-density lipoprotein cholesterol [HDL-C]: total cholesterol ratio) improved in the ginger group compared to the placebo group. Therefore, ginger was not only successful in suppressing inflammatory cytokine production but also in increasing insulin sensitivity and decreasing blood lipid levels, perhaps through modulating the activity of lipoprotein lipase.

Obesity is characterized by elevated levels of pro-inflammatory markers and is often associated with the development of MetSyn [295]. Breast neoplasms and MetSyn [296] have been associated with inflammation [295]. Thus, obese women with breast neoplasms were randomized for 6 weeks to receive either four 750 mg capsules/day of ginger or placebo to investigate ginger’s anti-inflammatory effects [78]. Those who received ginger had a significant decrease in CRP and IL-10 compared to the placebo group. The ginger group also had reductions in insulin, glucose, insulin resistance, LDL-C, and triglycerides and increases in HDL-C and HDL-C/LDL-C compared to baseline. Therefore, ginger supplementation successfully modulated both inflammatory and metabolic biomarkers, suggesting mitigation of inflammation and MetSyn severity. Since these biomarkers are linked to the development of breast neoplasms, ginger supplementation may have a protective effect against this as well.

Chemotherapy induces high oxidative stress, which causes adverse effects (e.g., nausea and vomiting), decreasing its effectiveness [297]. The oxidative stress occurs with decreased activity of antioxidant enzymes (i.e., SOD and catalase) and the increased production of peroxidation products (e.g., MDA) [298]. Animal studies have illustrated that ginger supplementation can increase catalase and SOD activity [299], protecting against oxidative stress. Thus, newly diagnosed cancer patients receiving moderate-to-high emetogenic chemotherapy were randomized to receive either 20 mg/day of ginger or placebo, starting from 3 days before beginning chemotherapy until 64 days later [79]. At the end of the intervention, SOD, catalase, glutathione peroxidase, and blood glutathione significantly increased, while the oxidative stress markers MDA and nitric oxide significantly decreased in the ginger group compared to placebo. These results demonstrate ginger’s effectiveness for patients receiving chemotherapy by improving antioxidant levels and decreasing markers of oxidative stress with no adverse effects.

TB is an infectious disease with significant global mortality, characterized by increased ROS production [300] and subsequent lipid peroxidation and tissue damage [301] mediated by inflammatory cytokines, especially TNF-α [302]. Thus, category one TB patients were randomized for 30 days to receive either 250 mg ginger extract twice/day or placebo in combination with standard treatment [80]. At the end of the treatment period, TNF-α, MDA, and ferritin significantly decreased in both groups, but more so in those taking ginger. Since these markers are normally elevated in TB and are thought to be involved in patients’ clinical deterioration [303], this suggests that ginger shows some potential as an adjunct anti-inflammatory therapy for TB.

Ginger has also successfully modulated TNF-α in knee OA, where the pain is caused by inflammation of the joint due to the overproduction of synovial cytokines. Older patients (50-70 years of age) with knee OA were randomized for 3 months to receive either 500 mg of ginger twice/day or placebo [81]. After treatment, those taking ginger saw a significant decrease in TNF-α and IL-1β compared to placebo, showing once again its ability to mitigate inflammatory cytokine production in a chronic condition.

#### 3.2.9. Hydrolyzed rice bran (rice bran arabinoxylan complex or biobran)

Rice bran contains a multitude of macronutrients, compounds, elements, and co-factors that have been shown to activate immune cells in addition to providing broad nutritional support [304,305]. More specifically, hydrolyzed rice bran (i.e., rice bran arabinoxylan complex) is an oligosaccharide complex produced through hydrolysis of rice bran hemicellulose B by *Lentinula edodes*, *Coriolus versicolor*, and *Schizophyllum commune* mycelia enzyme activity principally consisting of arabinose and β-1,4-xylopyranose moieties with lesser quantities of other polysaccharide chains, including beta-glucans [306]. Hydrolyzed rice bran has been found to demonstrate immunomodulatory activity *in vivo* in mice and humans and *in vitro* in cancer cell lines [307-309]. Other studies have also shown that varying hydrolyzed rice bran concentrations enhance macrophage phagocytic activity, the activities of IFN-γ, TNF-α, and IL-6, and modulation of NKCA [309-311].

Diseases such as multiple myeloma are categorized by diminished immune capacity. This process is partially facilitated by reduced DC count [312], which leads to decreased NKCA, as DCs activate NK cells [313]. Multiple myeloma patients also exhibit Th2 dominance and suppression of Th1 cytokines [314]. Thus, multiple myeloma patients were randomized for 3 months to receive either 2 g/day of hydrolyzed rice bran or placebo to determine its effects on multiple immune parameters [82]. After 1 and 2 months of treatment, NKCA significantly increased above the baseline level in the hydrolyzed rice bran group with no significant change in the placebo group. After 3 months of treatment, peripheral myeloid and plasmacytoid DCs significantly increased in the hydrolyzed rice bran group with no change in the placebo group. At baseline, most Th2 cytokines (i.e., IL-4, IL-5, IL-6, and IL-13) were elevated in the multiple myeloma patients, compared to healthy controls, and most patients had a Th1/Th2 score <1.0, suggesting Th2 dominance. After 1 month of treatment, Th1 cytokines (i.e., IL-1β, IL-12, IL-17, and TNF-α) significantly increased in the hydrolyzed rice bran group compared to baseline, and after 3 months, IL-12, IL-17, IFN-γ, and TNF-α increased. Th2 cytokines (IL-5 and IL-6) also increased in the hydrolyzed rice bran group after 1 month compared to baseline. After 3 months, IL-4, IL-6, IL-9, IL-10, and IL-13 increased compared to placebo, while IL-10 was significantly reduced in the placebo group. Therefore, hydrolyzed rice bran treatment elevated NKCA and DC counts and Th1 cytokine levels, which were all impaired at baseline, suggesting promise for multiple myeloma.

Nonalcoholic fatty liver disease (NAFLD) represents a spectrum of diseases characterized by hepatic fat accumulation [315] and occurs in roughly 20-30% of the population [316,317]. At present, NAFLD has no efficacious standard treatment, but symptom management is attempted with interventions aimed primarily at weight (fat) loss [315]. Two separate rat studies showed that hydrolyzed rice bran improved various biomarkers after d-galactosamine-induced acute liver disease, a model of hepatitis in humans [318,319]. The protective mechanism was mediated partially by downregulation of IL-18 in the first study [318] and by suppression of NF-κB and inhibition of CD14+ mRNA in the second study [319]. Thus, hydrolyzed rice bran was evaluated on complete blood count, liver enzymes, lipids, oxidative stress markers, cytokines, and growth factors in adults with NAFLD in a 90-day randomized, double-blind placebo-controlled trial [83]. Twenty-three adults with NAFLD were enrolled and randomly assigned to one of the two study conditions (*n*=12 hydrolyzed rice bran and *n*=11 placebo) and consumed 1 g/day of either compound. Alkaline phosphatase significantly decreased in the hydrolyzed rice bran group compared to the placebo. Percent monocytes, percent eosinophils, IFN-γ, and IL-18 significantly increased in the hydrolyzed rice bran group compared to the placebo group. Other improvements were noted for platelets, neutrophils, neutrophil-lymphocyte ratio, γ-glutamyl transferase, and 4-hydroxynonenal in the hydrolyzed rice bran group. Thus, hydrolyzed rice bran showed beneficial effects on several biomarkers that add to its known immunomodulatory activities, which may be promising for people with NAFLD.

While ART for HIV+ patients has extended their lifespan, it has been associated with inflammation, presumably related to overstimulation of CD8+ T cells [320,321]. Recently, the CD4+/CD8+ ratio has shown clinical utility in assessing immune activation and chronic inflammation [322,323]. A persistent inverted ratio (<1.0) is indicative of immunosenescence and is independently associated with markers of age-associated diseases such as atherosclerosis and renal impairment [324-326]. Given that nearly 80% of the HIV population on ART are below this clinical threshold of 1.0 [327-329], modifying the ART regimen to increase this ratio has proven to be challenging. However, hydrolyzed rice bran blocked HIV-1 replication by inhibiting p24 antigen production in a dose-dependent manner [307], warranting investigation of the effect of hydrolyzed rice bran on the CD4+/CD8+ ratio. Thus, hydrolyzed rice bran was evaluated on immune, hepatic, and renal function in HIV+ individuals for 6 months in a randomized, double-blind placebo-controlled trial [84]. Forty-seven HIV+ individuals on stable ART were enrolled and randomly assigned to one of the two study conditions (*n*=22 hydrolyzed rice bran and *n*=25 placebo) and consumed 3 g/day of either compound for 6 months. No side effects were reported, and liver and kidney markers remained nearly completely within normal limits. The percentage change in CD4+ T cells was similar for the placebo and hydrolyzed rice bran groups at 6 months follow-up, but the percentage change in CD8+ T cell count significantly decreased from baseline to 6 months in the hydrolyzed rice bran group, whereas it increased in the placebo group. The CD4+/CD8+ ratio improved clinically in the hydrolyzed rice bran group to over 1.0 at 6 months, whereas it declined in the placebo group to 0.72. Thus, hydrolyzed rice bran has been shown to demonstrate the following: (1) optimization of NKCA against cancerous and virus-infected cells, (2) enhancement of T and B cell counts; (3) proliferation of DCs; (4) regulation of cytokines and IL; (5) anti-inflammatory and antioxidant net effects; (6) no known adverse effects; and (7) modulation of NK, B, and T cells and global immune system function in a dose-dependent response in multiple subject groups (i.e., animal and human).

#### 3.2.10. Isoflavones (i.e., primarily daidzein and genistein)

Isoflavones, such as daidzein and genistein, are phytochemicals found in soy with known anti-inflammatory properties and have been therapeutic in cancer, obesity, and CVD [330]. Inflammatory pathways are linked to carcinogenesis and progression, and cancer patients often have elevated levels of pro-inflammatory cytokines (TNF-α, IL-6, and IL-1β) and expansion of regulatory cells (myeloid-derived suppressor cells and Tregs) [331]. Thus, to investigate the effects of soy isoflavones on prostate cancer, patients were randomized for 8 weeks to receive 2 slices/day of one of two soy bread formulations, one with 34 mg isoflavones/slice or one containing ground almonds [85]. Ground almonds contain B-glucosidase, which is hypothesized to increase the bioavailability of the isoflavones [332]. After treatment, Th1 cytokines (IL-1β, TNF-α, and IFN-γ) and myeloid-derived suppressor cell-associated cytokines (IL-6, IL-13, IL-10, granulocyte colony-stimulating factor, granulocyte macrophage colony-stimulating factor [GM-CSF], macrophage colony-stimulating factor, and VEGF) decreased in both groups. The percentage of CD56+ NK cells increased and Tregs and myeloid-derived suppressor cells decreased with no significant differences between groups. Thus, both kinds of soy bread reduced cell counts and subsequent production of cytokines linked to inflammation and cancer progression.

Obesity and MetSyn are characterized by low-grade inflammation and elevated pro-inflammatory cytokines, such as TNF-α, IL-6, and PAI-1 [333]. TNF-α is reported to inhibit the expression of plasma adiponectin [334] and induce PAI-1 [335]. Therefore, the elevation of TNF-α has a trimodal effect on inflammation. Obese subjects with MetSyn were randomized for 12 weeks to receive six 2 g capsules/day of soybean leaf extract or a placebo [86]. After treatment, the soy group saw significant decreases in TNF-α, PAI-1, and IL-6 compared to baseline and placebo. In addition, insulin resistance and free fatty acid level decreased in the soy group, showing an improvement in hyperglycemia and insulin sensitivity. Thus, soy isoflavones successfully alleviated low-grade inflammation from cytokine production, likely resulting in a decreased severity of insulin resistance.

#### 3.2.11. Mistletoe

Mistletoe (*Viscum album)* extracts have been used as complementary therapies to treat chronic disease due to their immunomodulatory properties. The three components of mistletoe (lectins, alkaloids, and viscotoxins) are hypothesized to drive the immunomodulatory effects [336], but the mechanisms behind their action are not fully understood. However, these components have been found to enhance immune responses in cancer, perhaps through increases in NKCA, as NK cells have been found to counteract tumor growth in humans [337]. In one study, tumor patients received subcutaneous treatment with aqueous mistletoe extract (15 ng mistletoe lectin/0.5 mL) twice/week for 48 weeks [87]. After treatment, NK cells significantly increased, while T lymphocytes (CD3+), cytotoxic T cells (CD8+), Th cells (CD3+CD4+), and T suppressor cells (CD3+CD8+) increased non-significantly. After discontinuing treatment for 6 weeks, most of the cell counts dropped to approximately baseline values, but NK cell count remained elevated. The treatment was well-tolerated by all patients. Therefore, mistletoe extract has the potential to improve cellular immunity in tumor patients, potentially mitigating tumorigenesis.

Cancer is generally immunosuppressive, and surgery and chemotherapy for the treatment of cancer also have immunosuppressive effects. For example, surgery can often induce NK cell suppression [338], promoting tumor cell growth and possibly generating new metastases [339]. Thus, patients undergoing CRC resection were randomized to receive either an infusion of 5 mg/mL mistletoe extract during the administration of general anesthesia or no mistletoe treatment to determine whether perioperative mistletoe supplementation would reverse the suppression of NKCA [88]. At 7 days postoperation, NKCA had risen above the baseline value in the mistletoe group, whereas it decreased significantly in the control group and remained below baseline until the 7^th^ day. Therefore, perioperative supplementation with mistletoe extract was successful in combating post-operative NK cell suppression in cancer patients.

In a similar study, patients with various gastrointestinal cancers were randomized to receive either 6 vials/week of 60 mg/mL aqueous mistletoe extract or placebo for 2 weeks preoperatively and 2 weeks postoperatively [89]. Th cell (CD4+) count significantly increased in both 1 day and 2 weeks postoperation in the mistletoe group, while this number decreased in the placebo group. T suppressor cell (CD8+) count significantly decreased at 2 weeks postoperation compared to a slight increase in the placebo group. IgA and IgM increased 2 weeks postoperation, while these values were unchanged in the placebo group. B cell count gradually increased in the mistletoe group, while it significantly declined in the control group 1 day postoperation. According to the Karnofsky Performance Scale Index, overall health status increased significantly in the mistletoe group and decreased significantly in the placebo group, while the reverse was true for scores on the Index’s anxiety scale. Therefore, mistletoe treatment appeared to enhance post-operative immune function and quality of life.

Similarly, radiation and chemotherapy can have adverse effects on WBC microcirculation, negatively impacting overall health [340]. Thus, patients with larynx and pharynx carcinoma were randomized for 12 weeks to receive either subcutaneous mistletoe extract or no additional treatment on top of their standard radiotherapy and chemotherapy to evaluate whether treatment with mistletoe extract could mitigate these effects [90]. Patients were assessed before surgery, before the start of radiation (1^st^ week), after the start of radiation (3^rd^ week), and at the 6^th^ and 9^th^ weeks. Immediately after the start of radiation, ICAM-1, the number of adhering WBC on the derma near the tumor, and the number of migrating WBC from the derma near the tumor significantly decreased in both groups. However, these decreases were significantly less severe in the mistletoe group. Furthermore, after the 6^th^ and 9^th^ weeks of treatment, the mistletoe group’s outcomes increased to near baseline levels and were significantly higher than those in the control group, which remained suppressed. Therefore, although it is difficult to avoid the initial immunosuppression at the onset of radiation, mistletoe supplementation could mitigate the suppression and aid in its nearly complete recovery within 6 weeks after its start. Overall, mistletoe supplementation appears effective in treating immunosuppression both from cancer itself and various cancer treatments, making it a potentially useful adjunct oncological therapy directly targeting various components of immune function.

#### 3.2.12. Resveratrol

Resveratrol is a polyphenol found in berries, grapes, and nuts [341] and has been found to decrease oxidative stress [342]. Male T2DM patients with hypertension were randomized to receive 350 mg/day for the first 6 months and 700 mg/day for the subsequent 6 months of either conventional grape extract without resveratrol, grape extract with resveratrol, or a placebo of maltodextrin [91]. At the end of the study, IL-6 significantly decreased only in the grape extract with resveratrol group. In the placebo group, adiponectin and IL-10 significantly decreased, while the IL-6/IL-10 ratio increased. The transcript levels of several pro-inflammatory mediators (TNF-α, IL-8, and IL-1β) significantly downregulated in the grape extract with resveratrol group compared to placebo. In addition, the level of TNF-α in PBMCs was significantly positively correlated with mRNA levels of TNF-α, IL-1β, and NFKBIA gene in the grape extract with resveratrol group. Serum IL-6 was negatively correlated with adiponectin and PBMC expression of IL-1β. Therefore, supplementation with resveratrol downregulated the expression in PBMCs of some inflammatory cytokines contributing to inflammation in T2DM.

Patients with stable CAD were randomized to participate in the identical previous study design by the same group of researchers [92]. In the placebo group, adiponectin and IL-10 significantly decreased, and PAI-1 significantly increased. In the grape extract with resveratrol group, CRP non-significantly decreased in a dose-dependent manner at both 6 and 12 months, and adiponectin significantly increased, being higher than the placebo group at 12 months. In addition, PAI-1 significantly decreased in the grape extract with resveratrol group. Since adiponectin protects against atherosclerosis and coronary lesions [343] and has been found to be suppressed in individuals with CAD [344], these findings are especially noteworthy. Adiponectin has also been found to be inversely related to PAI-1 in obese patients [345], so these results are consistent. In addition, adiponectin has been found to inhibit pro-inflammatory signaling in PBMCs [346], which is consistent with the decrease in IL-6 in the previous study and has important implications for decreasing CAD risk in this study. Overall, resveratrol supplementation appears to successfully increase the anti-inflammatory hormone adiponectin, thus mitigating inflammation, and decreasing CAD risk and severity of T2DM.

#### 3.2.13. Shiitake mushroom and its derivatives

Lentinan is a beta-glucan from the shiitake mushroom found to support T cell-dependent immunity [347] and induce the Th1 response, while downregulating the Th2 response [348]. Downregulation of the Th2 response is useful in oncology, as it has been shown that Th2 cytokine production modulates the immune response to malignant tumor growth [349] and that cancer patients exhibit Th2 dominance [350]. Thus, lentinan supplementation was investigated for its effects on Th1/Th2 balance in patients with digestive cancer [93]. Subjects received 2 mg every other day of intravenous lentinan 3 times leading up to tumor resection surgery. After treatment, the percentage of CD4+ T cells producing IFN-γ was significantly higher, and the percentage of CD4+ T cells producing IL-6 and IL-4 was significantly lower. The ratio of the percentage of IL-4 producing T cells after/before treatment was negatively correlated with that of the percentage of IFN-γ producing T cells after/before treatment and positively correlated with that of the percentage of IL-6 producing T cells after/before treatment. Eight out of 28 patients were considered treatment responders (quantified by a ratio over 2.0 for the percentage of IFN-γ producing T cells after/before treatment, i.e., more than double the percentage from baseline to the end of treatment). Ten patients had unresectable tumors and were given lentinan treatment after surgery (2 mg once/week) along with chemotherapy. Out of these 10, five were responders and had significantly longer survival time than the five non-responders. Overall, this study shows that lentinan treatment shifts the immune response toward Th1 dominance, as shown by the increases in IFN-γ production and decreases in IL-4 and IL-6 production. Furthermore, Th1 dominance was associated with higher survival time in those responders with unresectable tumors. Therefore, lentinan supplementation shows promise as an anti-tumor agent.

In another study, non-small cell lung cancer patients undergoing chemotherapy were either given an intramuscular injection of 4 mg/day of lentinan in addition to chemotherapy or chemotherapy only for 12 weeks [94]. CD3+CD56+ NK cells and CD3+CD8+ and CD3+CD4+ T cells significantly increased in the lentinan group compared to controls. Treg induction decreased along with significant decreases in IL-10 and TGF-b1 and increases in IFN-γ, TNF-α, and IL-12. In addition, the dominance from Th2 to Th1 response shifted. Therefore, lentinan appears to enhance cellular and anti-tumor immunity, while decreasing the immunosuppressive effects of Tregs. Overall, lentinan supplementation appears beneficial in multiple forms of cancer.

Active Hexose Correlated Compound (AHCC) is another immunomodulatory supplement derived from shiitake mushrooms. It has been found to modulate NKCA against infected cells [351], augment T cell function [352], and promote Th1 mediated immunity [353]. Peritoneal and epithelial ovarian cancer patients were randomized to receive either two capsules 3 times/day containing 500 mg AHCC or placebo throughout six cycles of chemotherapy [95]. At the sixth cycle of chemotherapy, CD8+ T cell lymphocytes were significantly higher in the AHCC group compared to placebo. Therefore, AHCC supplementation likely enhanced the immune response against tumor cells, which correlates with overall, progression-free survival [354].

## 4. Discussion

With the rising burden of chronic diseases and the acute threat of infectious agents, supporting immune function and simultaneously modulating inflammation have become areas of great interest among medical providers, researchers, and consumers. Although the maintenance of optimal immunity is influenced by numerous factors including sleep, physical exercise, and avoidance of toxins such as alcohol and tobacco, dietary supplementation has emerged as an actionable target for immunomodulation and counteracting chronic inflammation.

While a generally healthy diet adequate in macronutrients and rich in micronutrients forms the foundation for a functional immune system, targeted dietary supplementation tailored to the immunological status and specific health condition of an individual has shown promise in improving immune functioning and reducing inflammation. This narrative review of clinical trial data suggests that nutrients and phytonutrients are capable of significantly modulating immune function and reducing inflammation according to multiple biomarkers in different populations of adults with varying health conditions. These health conditions studied include HIV, autoimmune conditions, cancer, pulmonary diseases, and MetSyn, among others. The nutrients and phytonutrients of interest in these varied conditions are summarized below.

In HIV, ALCAR supplementation may exert a protective effect on mitochondrial and immune function [6]. Likewise, hydrolyzed rice bran has been studied in many clinical trials, demonstrating optimization of NKCA, enhancement of immune cell counts, regulation of cytokines and growth factors, anti-inflammatory and antioxidant activity, and modulation of global immune system function [84]. Another potential adjunct therapy is α-lipoic acid, which has been shown to enhance the lymphocyte proliferative response, possibly due to an increase in glutathione [13]. This targets the deficit in glutathione and impaired lymphocyte function, which are characteristic of HIV. However, no changes in CD4+ and CD8+ T cells were noted. NAC is another potential therapy acting through restoration of glutathione levels and immune function, regardless of treatment with ART or HAART [16,17]. Selenium intake also appears to suppress the viral burden of HIV-1, allowing for improvements in CD4+ T cell count in responders who achieve elevated serum selenium concentrations [23]. Successful restoration of peripheral blood lymphocyte viability and reduction in apoptosis has been demonstrated with alpha-tocopherol supplementation [32], while zinc supplementation was able to provide protection against lymphopenia and subsequent infections despite persisting viral load [54]. Many of these dietary supplements also showed higher adherence than standard therapy, making them a well-accepted adjunct to the conventional medical treatment regimen.

Many autoimmune diseases have also seen benefit from dietary supplementation. In lupus, NAC appears to be an effective therapy by increasing glutathione and restoring double-negative T cell function and Treg count [18]. Likewise, Vitamin D can restore B and T cell homeostasis in lupus by increasing Tregs and decreasing Th1, Th17, and memory B cells [40]. Antiphospholipid syndrome is often found in patients with lupus, although it can occur independently. CoQ10 appears to be effective in decreasing inflammation and severity of thrombosis in antiphospholipid syndrome, making it another potentially useful adjunct therapy [12]. In RA, black seed oil has been shown to decrease WBC count to within a normal level and increase blood glutathione level [65]. These changes in blood markers were accompanied by improvements in clinical symptoms, such as decreases in morning stiffness and the number of swollen joints. In fibromyalgia, CoQ10 shows promise as an adjunct anti-inflammatory, although it is likely insufficient as a sole therapy considering the small symptom reduction that was demonstrated [11]. MS is another autoimmune condition that may benefit from supplementation. Aloe polysaccharides [57], CoQ10 [10], curcumin [68,69], boswellic acids [72], lipoic acid [15], and high-dose Vitamin D [39] have all shown benefit in decreasing inflammation and improving the overall immunomodulatory profile in patients with MS. However, despite successful decreases in inflammation and imaging outcomes, some treatments such as boswellic acids were not entirely without side effects, causing gastrointestinal distress in a portion of patients. High-dose Vitamin D was also used in Addison’s patients to restore Vitamin D status, with resulting regulation of activated T cells and monocytes and a decrease in expression of the MHC isotype that facilitates the autoimmune reaction on late-activated T cells [41]. Likewise, in Crohn’s disease, Vitamin D successfully reduced the production of inflammatory cytokines [42]. However, the effect was not specific, but was an overall general reduction in cytokine production. Finally, Vitamin B12 supplementation can be used to treat immunodeficiencies in pernicious anemia, where it was found to restore function of the complement system and boost humoral immunity by restoring immunoglobulin concentrations [182].

Nutrients and phytonutrients have also shown promise in the adjunct treatment of various cancers. For example, Vitamin D exhibited increased intratumoral T cell infiltration and a reduction in HNSCC recurrence rate [43]. Vitamin D may also decrease colonic inflammation, reducing the risk of CRC neoplasms [46]. Likewise, ginger supplementation displayed potential for decreasing the risk of CRC development through anti-inflammatory mechanisms and was effective for patients receiving chemotherapy by improving antioxidant levels and decreasing markers of oxidative stress with no adverse effects [355]. Other digestive tract tumors have been treated with aged garlic extract [76], mistletoe extract [89], and beta-glucans [93], which have independently shown effects in combating immunosuppression and improving anti-tumor response. Beta-glucans have also been studied in breast cancer [59] and non-small cell lung cancer [94] with similar effects, while mistletoe extract appears effective in modulating immunosuppression both from cancer itself and from various cancer treatments [90]. Prostate and ovarian cancers have also shown benefit from dietary supplementation. Soy has shown anti-inflammatory properties and a decrease in progression of prostate cancer [85]. In ovarian cancer, AHCC may enhance the anti-tumor immune response, which correlates with overall survival in peritoneal and ovarian cancer patients [95]. AHCC also displayed potential benefit in the prevention of cervical cancer [356]. Finally, hydrolyzed rice bran in multiple myeloma has shown promise through elevation of NKCA and DC counts and Th1 cytokine levels, which were impaired at baseline [82].

Dietary supplementation has also been studied in various pulmonary and allergic conditions. In seasonal upper respiratory infections, garlic supplementation shows promise as an immunomodulator with noticeable decreases in symptom severity [357]. Zinc supplementation in the deficient elderly was shown to improve inflammatory markers and decrease infection risk [358]. On the contrary, Vitamin D supplementation can prevent seasonal fluctuations in Vitamin D status, but it has not demonstrated efficacy in preventing seasonal illness in healthy individuals, perhaps due to already optimized immune function [359]. In children with RRTI, treatment with beta-glucans improved innate and adaptive immunity, with decreases in respiratory morbidity and no adverse effects [58]. Another infectious pulmonary condition, TB, has been theorized to benefit from anti-inflammatory food compounds. Both ginger [80] and selenium [26] have shown some promise in exerting anti-inflammatory effects, decreasing oxidative stress, and improving immune function in this population with often insufficient nutritional intake. Among cystic fibrosis patients, high-dose NAC supplementation effectively augments glutathione level and mitigates inflammation [19]. Airway inflammation was also reduced by EΩ3FA supplementation in patients with cystic fibrosis [33]. In addition, EΩ3FA supplementation can improve asthma severity through reduction of airway inflammation [34]. Airway inflammation in asthma is also the target of gamma-tocopherol [49-51] and selenium [27] supplementation, both of which can reduce airway reactivity. In allergic conditions, beta-glucans may prove useful through a shift in the immune system toward a Th1-mediated response [61]. Similarly, black seed oil supplementation enhances the Th1 response and shows potential for alleviating the severity of allergic symptoms and possibly decreasing the risk of developing atopic dermatitis in infants [66,67].

Dietary supplements also demonstrate efficacy in the treatment of MetSyn and related disorders. Many of these enhancements are thought to be mediated by reductions in chronic low-grade inflammation and improvement in insulin sensitivity, endothelial function, and lipoprotein profile. Beneficial nutrients and phytonutrients include bilberry [64], garlic [75], ginger [295], soy [86], α-lipoic acid [14], zinc [55], and resveratrol derivatives [360]. The mechanisms of action of these nutrients also help the common comorbidities found in MetSyn, including CVD and T2DM. Specific benefit in T2DM has been found with curcumin [71], ginger [77], resveratrol [91,92], selenium [25], zinc [55], Vitamin D [47], and alpha-tocopherol [29-31], with noted improved inflammation through various mechanisms. Several of these compounds, including ginger [77] and Vitamin D [47], have also demonstrated better insulin sensitivity. In NAFLD, hydrolyzed rice bran may exert immunomodulatory effects as noted by positive changes on a number of biomarkers [83]. The increased cardiovascular risk conferred by MetSyn may also be mitigated through the supplementation of nutritional compounds including poly- and monounsaturated fatty acids [361], garlic [74], and Vitamin C [362,363]. Vitamin C has demonstrated additional potential in improving the smoking-mediated risk of CVD [364], while boswellic acids may improve ischemic stroke risk and outcomes by mitigating the release of inflammatory mediators [73]. CKD, another common comorbidity in MetSyn, may get better with supplementation. NAC produced a reduction in inflammation and cardiovascular comorbidities in CKD patients receiving continuous ambulatory peritoneal dialysis [20]. Likewise, EPA and DHA supplementation appeared to decrease inflammation in patients with low-grade inflammation from CKD [36], while zinc supplementation resulted in decreased oxidative stress and normalization of T cell function [53]. Finally, high-dose Vitamin D was studied in dialysis patients with potential improvement in Vitamin D metabolism and an overall anti-inflammatory effect [48].

In addition to the previously mentioned conditions, various nutrients and phytonutrients have also been investigated in the treatment of Alzheimer’s disease, OA, lead poisoning, burns, sickle cell patients, and ICU patients. In Alzheimer’s disease, aloe polymannose multinutrient complex improved cognitive function through reduction in inflammation and endothelial dysfunction and modulation of lymphocyte activity [56]. In OA, the anti-inflammatory activity of both curcumin [70] and ginger [81] has proven beneficial in lowering inflammatory mediators, without the deleterious side effects on gastrointestinal and renal systems that are common with conventional NSAID therapy. An elevated blood level of lead has been successfully treated with NAC, which was shown to decrease blood lead content and improve antioxidant activity in a dose-dependent manner [21]. NAC has also been used in the treatment of burn patients, leading to a reduction in inflammation and in the required amount of inotropic and vasopressor drugs, perhaps through reduction in edema and fluid requirements [22]. In zinc-deficient sickle cell disease patients, zinc supplementation has shown antioxidant, erythropoietic, and immunomodulatory effects, while decreasing soluble adhesion molecule concentrations and infection incidence [52]. Finally, beta-glucans may serve as a potential therapy in critical illnesses by increasing cell-mediated immunity without causing additional inflammation [62].

Overall, the results of these clinical trials suggest promise for the immunomodulatory and anti-inflammatory effects of targeted dietary supplementation. High tolerability and minimal side effects of the nutrients and phytonutrients evaluated in these trials are especially encouraging. Many of these compounds also have a long history of culinary use, adding to the safety profile of long-term consumption, although doses greater than those found in culinary use may pose a different level of risk or consideration. Dietary supplementation also has the potential for a comparatively inexpensive means of immunomodulation, and the effector compounds are often phytochemicals found in plant foods that are otherwise healthful. However, given the significant immunomodulatory responses, caution must be exercised in the use of some of these supplements, particularly those that are fat soluble. In certain cases, a higher level or supraphysiological supplementation of nutrients may have negative effects on health. With some compounds such as selenium, a class of immunologic markers may be improved while others are worsened [365]. How to manage these heterogeneous effects and whether the net effect is beneficial in a particular health condition still necessitates further study.

In addition, some compounds only show benefit in individuals with deficiency. Dietary supplements can also exhibit a variety of interactions with both prescription and non-prescription medications through direct interaction or modulation of key enzymes implicated in drug metabolism. Thus, any addition of dietary supplements to a given daily regimen warrants careful review of existing medications.

Although many of these studies were well-designed randomized placebo-controlled trials, some design limitations exist. Some studies included very small sample sizes and the follow-up time in trials ranged substantially from hours to years, making the prediction of long-term effects of supplement use difficult for certain nutrients. While many trials found significant symptomatic improvement or modulation of disease activity, many studies solely measured immunologic markers without investigating other clinical endpoints. Thus, it is difficult to know whether changes in various immunologic markers would translate to clinically significant treatment effects. Due to these limitations, additional large studies are needed to investigate the safety and efficacy of many of these compounds in the treatment and prevention of various health conditions, with emphases on clinical endpoints and potential drug-supplement interactions, especially when combined with conventional therapy.

This type of narrative review has several inherent limitations. We did not synthesize or pool data for each nutrient or phytonutrient and condition. Thus, it is difficult to make any conclusions about the overall efficacy of these nutrients and phytonutrients. Furthermore, we did not compare the efficacy of different dosages among studies, so in most cases, the ideal dose of any supplement is not clear. It is also not certain how dosages may need to differ depending on the disease or disorder. We did not limit our review to a certain treatment duration, so it is difficult to determine how outcomes translate to improvements in lifespan, even for studies that evaluated a year-long intervention. Another limitation inherent to dietary supplement nutrient and phytonutrient trials includes differences in bioavailability and absorption of active ingredients, which are likely causative factors in producing significant outcomes. For example, curcuminoids are known to have very low naturally-occurring bioavailability and absorption and the way they are formulated in a test product could have dramatic effects on study outcomes [366]. In addition, for those nutrients with a recommended daily intake, such as vitamins and minerals, a failure to collect dietary intake data as part of an assessment protocol may change or inflate the true treatment effect, if subjects are consuming foods that also contain the treatment being evaluated.

Historically, plant compounds have served as an important source of therapeutic and pharmaceutical discovery and exhibit immensely complex multi-physiological and metabolic activity. This summary review highlights the evidence that nutrients and phytonutrients can modulate inflammatory and immunologic responses, with potential benefit for the adjunct treatment of a variety of conditions ranging from acute infection to malignancy. The results also suggest that outcomes of treatment may differ depending on the immunologic state of the subject, making individualization of the treatment regimen an important consideration. In addition, evaluation of risks and benefits for each patient and health status is prudent. Thus, dietary supplementation with key nutrients and phytonutrients may offer an avenue for improving immune function, with benefit in a chronic illness or acute infection.

### Conflict of interest

The authors declare that they have no conflicts of interest.
